# Targeted NUDT5 inhibitors block hormone signaling in breast cancer cells

**DOI:** 10.1038/s41467-017-02293-7

**Published:** 2018-01-17

**Authors:** Brent D. G. Page, Nicholas C. K. Valerie, Roni H. G. Wright, Olov Wallner, Rebecka Isaksson, Megan Carter, Sean G. Rudd, Olga Loseva, Ann-Sofie Jemth, Ingrid Almlöf, Jofre Font-Mateu, Sabin Llona-Minguez, Pawel Baranczewski, Fredrik Jeppsson, Evert Homan, Helena Almqvist, Hanna Axelsson, Shruti Regmi, Anna-Lena Gustavsson, Thomas Lundbäck, Martin Scobie, Kia Strömberg, Pål Stenmark, Miguel Beato, Thomas Helleday

**Affiliations:** 1 Science for Life Laboratory, Division of Translational Medicine and Chemical Biology, Department of Medical Biochemistry and Biophysics, Karolinska Institutet, Solna, SE-171 21 Sweden; 2 Centre de Regulació Genòmica (CRG), Barcelona Institute for Science and Technology, Barcelona, E-09003 Spain; 3 Universitat Pompeu Fabra, Barcelona, E-08003 Spain; 4 Department of Biochemistry and Biophysics, Stockholm University, Stockholm, SE-106 91 Sweden; 5 Uppsala University Drug Optimization and Pharmaceutical Profiling Platform, Department of Pharmacy, Uppsala University, Uppsala, SE-751 23 Sweden; 6 Chemical Biology Consortium Sweden, Science for Life Laboratory, Division of Translational Medicine and Chemical Biology, Department of Medical Biochemistry and Biophysics, Karolinska Institutet, Solna, SE-171 21 Sweden

**Keywords:** Breast cancer, Screening, Target validation, Drug discovery and development

## Abstract

With a diverse network of substrates, NUDIX hydrolases have emerged as a key family of nucleotide-metabolizing enzymes. NUDT5 (also called NUDIX5) has been implicated in ADP-ribose and 8-oxo-guanine metabolism and was recently identified as a rheostat of hormone-dependent gene regulation and proliferation in breast cancer cells. Here, we further elucidate the physiological relevance of known NUDT5 substrates and underscore the biological requirement for NUDT5 in gene regulation and proliferation of breast cancer cells. We confirm the involvement of NUDT5 in ADP-ribose metabolism and dissociate a relationship to oxidized nucleotide sanitation. Furthermore, we identify potent NUDT5 inhibitors, which are optimized to promote maximal NUDT5 cellular target engagement by CETSA. Lead compound, TH5427, blocks progestin-dependent, PAR-derived nuclear ATP synthesis and subsequent chromatin remodeling, gene regulation and proliferation in breast cancer cells. We herein present TH5427 as a promising, targeted inhibitor that can be used to further study NUDT5 activity and ADP-ribose metabolism.

## Abstract (web summary)

NUDIX hydrolases are an important family of nucleotide-metabolizing enzymes. Here, the authors identify potent, small molecule inhibitors of NUDT5, which is implicated in ADP-ribose and 8-oxo-guanine metabolism, and confirm its role in gene regulation and proliferation in breast cancer cells.

## Introduction

The NUDIX hydrolases are a core family of nucleotide-metabolizing enzymes that have critical roles in health and disease^1–3^. With a diverse network of substrates, NUDIX enzymes hydrolyze nucleoside diphosphates that are linked to a variable group (X), and contain the NUDIX box motif: GX_5_EX_7_REUXEEXGU, where X can be any residue and U represents a hydrophobic residue (usually Leu, Val, or Ile)^1,2^. The 22 different NUDIX enzymes have been implicated in a variety of biological processes^1,2,4^, including nucleotide pool sanitation and the efficacy of antimetabolite chemotherapeutics^5,6^. The best-characterized NUDIX enzyme, MTH1 (NUDT1, NUDIX hydrolase 1), is a sanitizer of the nucleoside triphosphate pool, responsible for degrading oxidized purine nucleotides to safeguard nucleic acid integrity^7–9^. Important roles for other NUDIX family members continue to be uncovered and highlight the essential role of sanitation enzymes in nucleotide biochemistry^10^.

Similar to MTH1, NUDT5 (NUDIX hydrolase 5 or NUDIX5) has been linked to key processes involved in nucleotide metabolism and cancer^11,12^. Two predominant substrates have been identified for NUDT5: 8-oxo-dGDP and adenosine 5′diphosphoribose (ADPR)^13–17^. While there is evidence that NUDT5 can hydrolyze 8-oxo-dGDP under basic conditions (pH ≈ 10)^13^, the physiological role of NUDT5 in 8-oxo-guanine metabolism has not been rigorously studied^13,18^. ADPR is an important signaling molecule in cells and is linked to the DNA damage response through the activity of poly(ADPR) (PAR)-related enzymes^19–21^. NUDT5 (and NUDT9) catalyze the breakdown of ADPR to ribose-5-phosphate (R5P) and adenosine 5′-monophosphate (AMP)^17,22^, which permits recovery of NAD^+^ pools after DNA damage and poly(ADPR) polymerase (PARP) activation^21^. In addition, NUDT5 was recently shown to be responsible for the production of PAR-mediated nuclear ATP and, thus, subsequent ATP-dependent chromatin remodeling and gene regulation following progestin or estrogen stimulation in breast cancer cells^23^.

Here we investigate the roles of NUDT5 in 8-oxo-guanine and ADPR metabolism. We demonstrate that NUDT5 poorly catalyzes the hydrolysis of 8-oxo-dGDP under physiological pH in vitro. Similarly, knockdown of NUDT5 fails to induce DNA damage or influence OGG1-specific lesions in DNA, suggesting that NUDT5 may be dispensable for maintaining genome integrity via 8-oxo-guanine sanitation. Instead, we provide additional support that NUDT5 is an integral component of ADPR metabolism. To further explore NUDT5 biology, we develop targeted NUDT5 inhibitors via a cellular thermal shift assay (CETSA)-guided screening funnel and utilize these compounds to study the role of NUDT5 in progestin-stimulated breast cancer cells. Lead compound, TH5427, is a versatile NUDT5 probe that can shed new light on nuclear ATP dynamics and ADPR-related metabolism in cells.

## Results

### NUDT5 is a regulator of ADPR metabolism

**Fig. 1:**
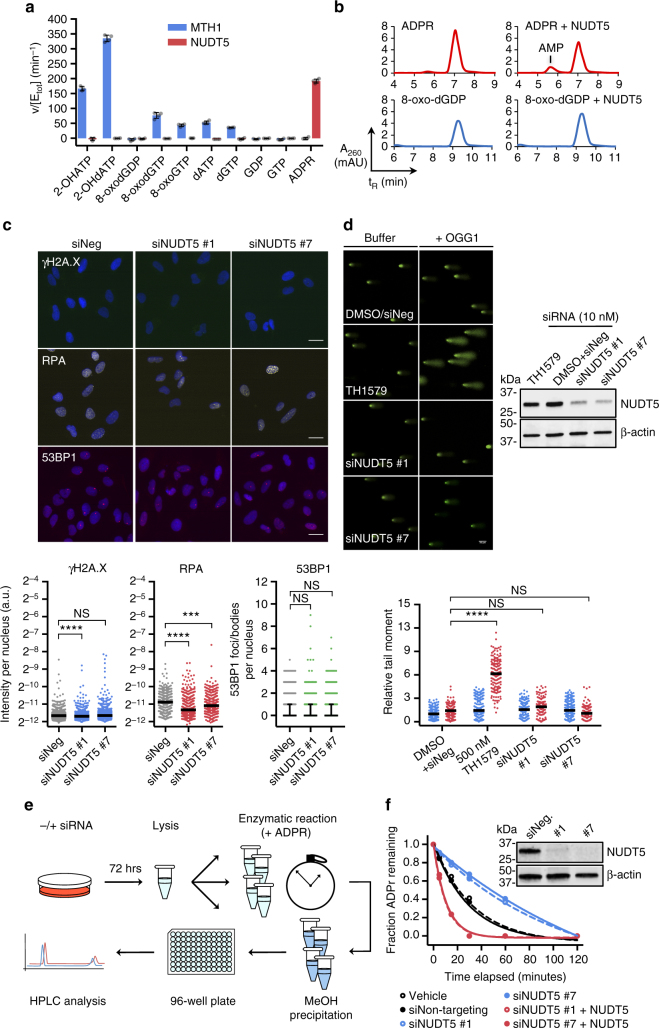
NUDT5 is a key regulator of ADP-ribose metabolism. **a** Hydrolysis of potential oxidized nucleotides and nucleotide-sugar substrates by MTH1 (blue) and NUDT5 (red), as measured by the enzyme-coupled malachite green assay (MG assay), at pH 7.5. A representative experiment (of *n* = 2) with mean ± SD and individual values of quadruplicate replicates is shown. **b** Representative HPLC traces of NUDT5-mediated ADPR (red) and 8-oxo-dGDP (blue) hydrolysis to AMP or 8-oxo-dGMP, respectively (*n* = 2). **c** Top: Representative, pseudo-colored immunofluorescence stainings for γH2A.X, chromatin-bound RPA, and 53BP1 in U-2 OS cells following treatment with negative control siRNA (siNeg) or NUDT5 siRNA (siNUDT5 #1, siNUDT5 #7) for 72 h. Scale bar = 20 µm. Bottom: Quantification of γH2A.X, chromatin-bound RPA, and 53BP1 staining intensity in cells representing three independent experiments (cells scored: γH2A.X, *n* = 871; RPA, *n* = 463; 53BP1, *n* = 981). Lines represent median fluorescence (and interquartile range for 53BP1); a.u. arbitrary units. NS not significant, ***p < 0.001, ****p < 0.0001; Kruskal-Wallis test). **d** Top: Representative, pseudo-colored images of treated U-2 OS cells following the modified alkaline comet assay and confirmation of NUDT5 knockdown with a cropped western blot. Scale bar = 50 µm. Bottom: Quantification of the tail moment (relative to the DMSO+siNeg, buffer-treated control) from two independent experiments performed in duplicate (minimum of 100 cells per slide per condition). Buffer-treated samples are shown in blue, OGG1-treated samples in red. Lines represent median tail moments. ****p < 0.0001 by Kruskal–Wallis test comparing OGG1-treated samples. **e** A graphical procedure depicting the analysis of ADPR hydrolysis in cell lysates by HPLC. **f** ADP-ribose hydrolysis in U-2 OS cells alone (black, solid and dotted), following depletion with two different NUDT5 siRNAs (blue, solid and dotted) or siRNA-treated cells complemented with purified NUDT5 protein (red, solid and dotted), as quantified by HPLC. A representative chromatogram is shown (of *n* = 2 independent experiments) and NUDT5 knockdown confirmation with cropped western blot

While both 8-oxo-dGDP and ADPR have been identified as potential NUDT5 substrates, experiments under physiological conditions have been largely absent from the scientific literature. To help clarify the preferred substrate(s) for NUDT5, we screened pertinent canonical and oxidized nucleotide species, as well as ADPR, at physiological pH (7.5) using an enzyme-coupled malachite green-based assay (herein called simply the MG assay, Fig. 1a) with purified human NUDT5 and MTH1 (Supplementary Fig. 1). Distinct from MTH1, NUDT5 had negligible activity against all tested oxidized and canonical nucleoside diphosphate and triphosphate species but catalyzed efficient turnover of ADPR. HPLC analysis confirmed the release of AMP as the expected product of NUDT5-mediated ADPR hydrolysis (while R5P is not detectable; Fig. 1b)^24^. Accordingly, no activity was observed with 8-oxo-dGDP by HPLC (Fig. 1b).

Furthermore, siRNA-mediated knockdown of NUDT5 alone did not appreciably induce DNA damage signaling in U-2 OS cells, as measured by multiple DNA damage markers (γH2A.X, 53BP1 and chromatin-bound RPA; Fig. 1c). In fact, γH2A.X and RPA signals generally decreased to some extent. In addition, genome integrity and OGG1-specific DNA lesions were unaffected by NUDT5 depletion in the modified alkaline comet assay (Fig. 1d), which was in stark contrast to treatment with the MTH1 inhibitor, TH1579 (karonudib)^25^. Altogether, these data suggest that (1) 8-oxo-dGDP is a poor substrate for NUDT5 at physiological pH and (2) that NUDT5 is not critical for oxidized nucleoside pool sanitation under these conditions.

We then used HPLC to monitor ADPR hydrolysis in U-2 OS cell lysates, which emulated the cellular context and was based on an earlier procedure performed in yeast^26^ (schematic illustration in Fig. 1e). Following depletion with NUDT5 siRNAs prior to cell lysis, ADPR hydrolysis was markedly attenuated compared to non-targeting siRNA controls. As expected, this could be rescued by the addition of recombinant NUDT5 protein (Fig. 1f), indicating that NUDT5 plays an important role in cellular ADPR turnover.

### Small molecule screening campaign for NUDT5 inhibitors

**Fig. 2:**
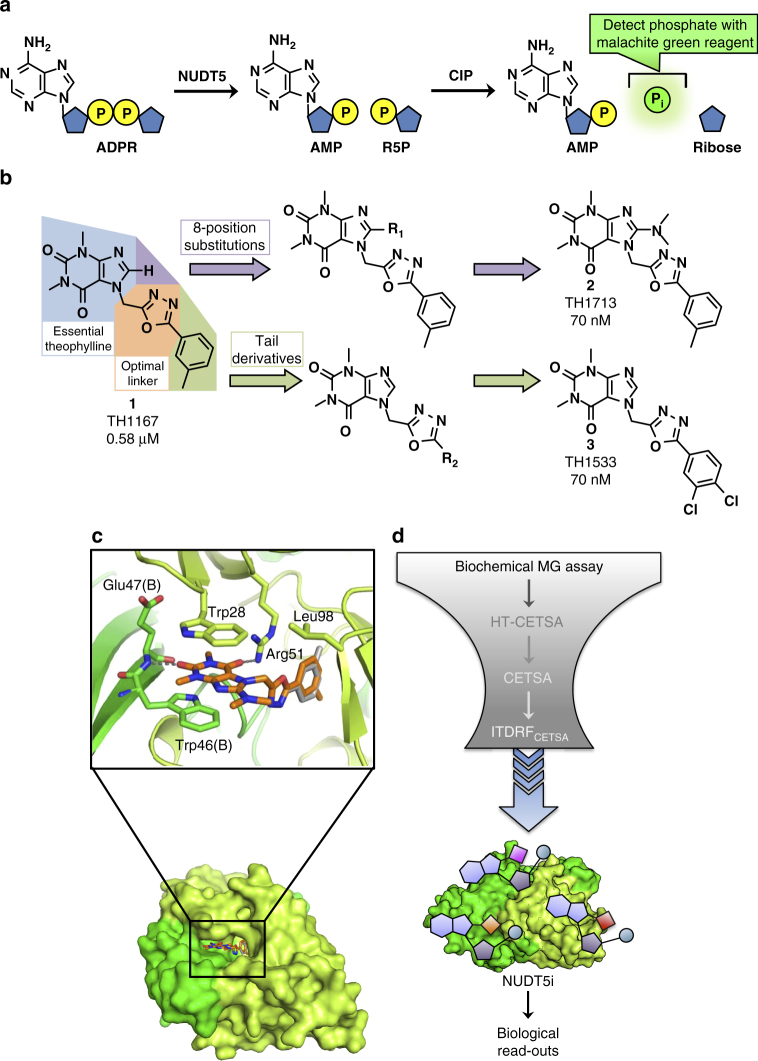
Development of potent NUDT5 inhibitors from a high-throughput screening campaign. **a** A representation of the enzyme-coupled malachite green assay (MG assay). ADPR is hydrolyzed to AMP and R5P by NUDT5, then R5P is converted to free inorganic phosphate detected by the malachite green reagent. **b** Preliminary structure–activity relationship study for determining where modifications are tolerated. Favorable optimization of TH1167 was performed by modification at the theophylline 8-position (purple) and tail position (green) of the molecule. MG assay IC_50_ values are reported below the chemical structures. **c** Structure of NUDT5 with inhibitor TH1713. Binding of TH1713 (orange) by NUDT5 dimer (chain A, limon green and chain B, green) through stacking between Trp28 of chain A and Trp46 of chain B and hydrogen bonds to the amide nitrogen of Glu47 and side chain of Arg51. An alternate conformation of the methylbenzene modality is modeled in gray. **d** NUDT5 inhibitor screening funnel optimized to select for target engagement in cells

We next sought to develop small molecule NUDT5 inhibitors to further probe the role of NUDT5 in ADPR metabolism. A high-throughput, screening-compatible NUDT5 inhibition assay was established using slight modifications to the aforementioned MG procedure (Fig. 2a)^15,27^. Briefly, ADPR, NUDT5 and calf intestinal alkaline phosphatase (CIP) were combined in a coupled enzymatic assay, as described in the online methods. In turn, ADPR was hydrolyzed to AMP and R5P by NUDT5, and the released R5P was further degraded to ribose and inorganic phosphate (P_i_) by CIP. Free P_i_ was subsequently detected using the malachite green reagent. The screen was conducted using a combination of the Chemical Biology Consortium of Sweden (CBCS) primary screening set and several commercial libraries from Enamine, TimTec, Maybridge and ChemDiv. In total, ~72 000 distinct chemical entities were screened for their potential inhibition of NUDT5 at a concentration of 10 µM (Supplementary Table 1, Supplementary Fig. 2a, b). Execution of this screening campaign was split between two different occasions. The screening performance in the two screening campaigns was excellent (average z´ factors of 0.87 and 0.85, Supplementary Fig. 2c), resulting in a hit limit as defined by the average and three standard deviations of the library compound responses of 10.1 and 18.4% inhibition, respectively (Supplementary Table 1, Supplementary Fig. 2a, b). This translated to 53 and 475 hits (0.3 and 0.8% hit rate, respectively), yielding 528 total hits from the 2 separate screening occasions.

### Preliminary optimization of hit compounds

Of the 528 hit compounds, 71 compounds possessed a theophylline group, which was identified as a key functional group for NUDT5 inhibition. The most potent compound, **1** (TH1167, MG assay IC_50_ = 0.58 µM, depicted in Fig. 2b), was selected for optimization in structure–activity relationship (SAR) studies. Preliminary medicinal chemistry efforts aimed to increase the potency of TH1167. Four major sites were identified for diversification: (1) the theophylline portion (blue), (2) the 8-position of the theophylline ring (purple), (3) the linker region (orange), and (4) the tail position of the molecule (green), depicted in Fig. 2b. The theophylline moiety of TH1167 was deemed necessary for compound activity due to the high number of hit compounds that possessed this functional group. Incorporation of additional functionality at the 8-position of the theophylline ring was well-tolerated and led to improved inhibitory activity. The 8-dimethylamino variant, **2** (TH1713, MG assay IC_50_ = 70 nM, Fig. 2b), had more than 8-fold greater potency over TH1167. While replacing the oxadiazole ring with other linker groups did not improve NUDT5 inhibition, derivatization of the tail or 5-position of the oxadiazole ring led to increased potency (Fig. 2b). The most active tail derivative, **3** (TH1533) incorporated the 3,4-dichlorobenzene group as the optimal tail and was equipotent with TH1713.

To put these inhibitors into perspective, we also tested compounds that were previously reported to inhibit NUDT5 and/or NUDT9 activity in HeLa cell extracts^21^, which included chelerythrine (a PKC inhibitor), LY294002 (a PI3K inhibitor), *p*-coumaric acid and flavone. None of these had any direct inhibitory effect on NUDT5 activity in the enzyme-coupled malachite green assay (IC_50_ values>100 µM; Supplementary Fig. 3).

### Cellular target engagement

Next, we sought to further characterize NUDT5 inhibitors with biophysical techniques to determine if they would bind the intended target in a cellular setting. Target engagement assays, such as the cellular thermal shift assay (CETSA), have allowed for unprecedented understanding of ligand-target interactions in live cells^28^. We first verified that the inhibitors could stabilize NUDT5 against thermal denaturation using differential scanning fluorimetry (DSF). Preliminary lead compounds TH1533 and TH1713 each resulted in a 5 °C shift in melting temperature (*T*_m_) at 20 µM compound concentrations (Supplementary Fig. 4a), indicating inhibitor binding.

To establish a protocol for cellular target engagement, intact HL-60 cells and cell lysates were subjected to a temperature gradient and soluble NUDT5 levels were assessed by western blot with thermostable SOD1 as a loading control (Supplementary Fig. 4b). For the purpose of screening, 83 °C was selected as the ideal temperature to evaluate NUDT5 engagement, as nearly all of the protein had aggregated at this temperature. The high temperatures that were required to induce NUDT5 unfolding were also found to compromise cell membrane integrity (Supplementary Fig. 4c), thus cells were washed twice with PBS in order to remove excess compound before the transient heating step. Without such additional precautions, the binding studies would be severely compromised by the leaking membranes^29^.

TH1167 was a potent inhibitor in vitro but did not show sufficient target engagement in cell lysates or intact cells at 83 °C (Supplementary Fig. 4d). Synthetic derivatives, TH1713 and TH1533, had more potent biochemical activity and could stabilize NUDT5 against thermal degradation in vitro; however, only TH1533 showed activity by CETSA in intact cells (Supplementary Fig. 4d). Encouraged by the result with TH1533, we sought to further optimize these compounds to increase NUDT5 target engagement under the described conditions. TH1533 and TH1713 both suffered from very poor solubility, which also necessitated further medicinal chemistry efforts.

### Structural insight to inhibitor design

To confirm the binding modality of the NUDT5 inhibitors and help guide further inhibitor development, NUDT5 was crystallized in complex with TH1713, and the structure was solved at 2.2 Å resolution (Fig. 2c, Supplementary Fig. 5 and Table 1). TH1713 occupies the same binding regions as ADPR within the active site of the NUDT5 dimer^30^, which is comprised of residues from chain A and chain B. The theophylline ring of TH1713 is anchored in place by two hydrogen bonds between its carbonyl oxygen atoms and the amide nitrogen of Glu47 (chain B), as well as the guanidinyl group of Arg51 (chain A). Pi stacking interactions between the theophylline ring and the indole rings of Trp28 (chain A) and Trp46 (chain B) further strengthen inhibitor binding. The oxadiazole group demonstrates variable water coordination and the 3-methylbenzene group extends into a hydrophobic cavity where it rotates between two alternate orientations. The 8-dimethylamino group on the theophylline ring extends out of the active site towards the solvent, which provides insight into the observed tolerability of modifications at this position.

**Table 1:** Crystallography data collection and refinement statistics (molecular replacement)

	NUDT5 TH1713	NUDT5 TH5427
*Data collection*		
Space group	C 1 2 1	C 1 2 1
Cell dimensions		
*a*, *b*, *c* (Å)	111.5, 39.3, 98.72	100.6, 40.1, 104.1
α, β, γ (°)	90, 122.2, 90	90, 113.4, 90
Resolution (Å)	41.8–2.2 (2.3–2.2)^a^	46.2–2.6 (2.8–2.6)^a^
*R*_sym_ or *R*_merge_	10.9 (96.5)	7.5 (59.9)
*I* /σ*I*	9.7 (1.4)	15.6 (2.5)
Completeness (%)	96.6 (93.2)	98.0 (95.6)
Redundancy	3.2 (2.8)	3.5 (3.6)
*Refinement*		
Resolution (Å)	41.8–2.2	46.2–2.6
No. reflections	18626	11883
*R*_work_/*R*_free_	18.0/23.8	19.6/25.5
No. atoms		
Protein	3037	3049
Ligand/ion	87	66
Water	174	46
*B*-factors		
Protein	46.3	57.4
Ligand/ion	49.4	81.2
Water	87.5	42.7
R.m.s. deviations		
Bond lengths (Å)	0.01	0.007
Bond angles (°)	1.5	1.3

^a^Data for each structure was collected from single crystals. Highest-resolution shell is shown in parentheses

### Utilizing progressive CETSA to drive compound optimization

A screening funnel was designed to prioritize compounds that engaged NUDT5 under progressively more stringent biological conditions (Fig. 2d). After first assessing biochemical inhibition of NUDT5 using the MG assay, compounds with an IC_50_<100 nM were tested for their ability to thermostabilize NUDT5 in cell lysates at single doses by high-throughput CETSA (HT-CETSA). This approach allowed for practical screening of inhibitors and quickly identified compounds that could bind to NUDT5 in a biologically demanding setting. Compounds that showed engagement in cell lysates were then evaluated at a single concentration to determine if they could engage NUDT5 in intact cells. Finally, isothermal dose-response fingerprint CETSA (ITDRF_CETSA_) was performed to establish which inhibitors could stabilize intracellular NUDT5 at the lowest concentrations. Relying on cellular target engagement assays to select our top compounds ensured that our medicinal chemistry campaign was not biased towards compounds that caused a predefined phenotypic response.

**Fig. 3:**
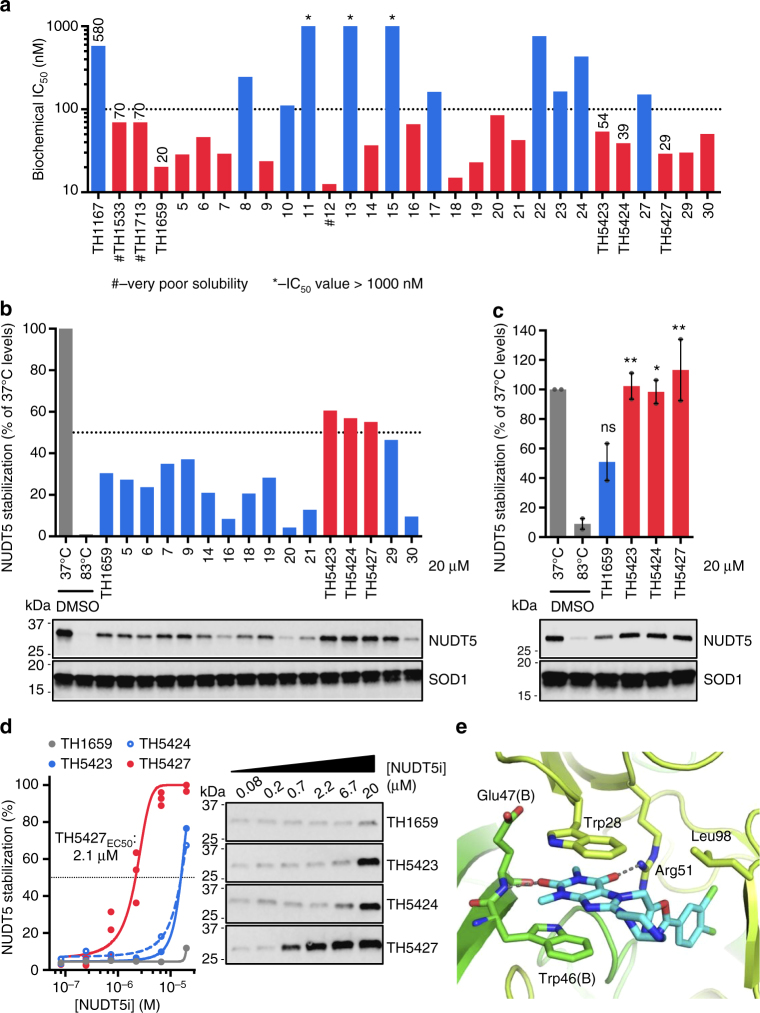
CETSA-guided screening distinguishes TH5427 as a lead NUDT5 inhibitor. **a** Biochemical characterization of compounds shown in Table 2 by enzyme-coupled malachite green assay (MG assay). Compounds of particular interest are labeled with their code names and MG assay IC_50_ values (nM). **b** Compounds with MG assay IC_50_ < 100 nM and reasonable solubility were tested by high-throughput CETSA (HT-CETSA) in HL-60 cell lysates. Values shown are relative to NUDT5 band intensity at 37 °C, normalized to SOD1 and a representative of two experiments. Cropped, representative blots are included. **c** Compounds with > 50 % stabilization in cell lysate CETSA were tested for target engagement at 20 µM with intact HL-60 cells by CETSA. Values are plotted relative to NUDT5 band intensity at 37 °C and normalized to SOD1. Data are means ± SEM and individual points from *n* = 2 experiments. Cropped, representative blots are shown. NS not significant, *p < 0.05, **p < 0.01; one-way ANOVA analysis. **d** HL-60 cells were treated with the same compounds from **c** but titrated by serial dilution from 20 µM to 0.06 µM for isothermal dose-response fingerprint CETSA (ITDRF_CETSA_). Data for TH5427 comprise data points from three independent experiments and representative experiments are shown for the remaining compounds. Representative, cropped blots are also shown. In all cases above, compounds colored in red progressed to the next stage of evaluation. **e** Structure of NUDT5 with TH5427 bound. Coordination of TH5427 (cyan) by NUDT5 dimer (chain A – limon green and chain B – green) via stacking interactions between Trp28 of chain A and Trp46 of chain B and hydrogen bonds to the amide nitrogen of Glu47 and side chain of Arg51

A focused library of NUDT5 inhibitors incorporating characteristics of both TH1533 and TH1713 was developed with the aim of improving inhibitor activity and solubility. Novel NUDT5 inhibitors are displayed in Table 2 with their corresponding MG assay IC_50_ values plotted in Fig. 3a (synthetic protocols can be found in the Supplementary Methods). While the combination of the 3,4-dichlorophenyl tail from TH1533 and the dimethylamino group of TH1713 was moderately active in the MG assay (**11**, MG assay IC_50_ = 0.11 µM), this compound also suffered from very poor solubility, which likely contributed to its compromised efficacy. Compound **13** also had major solubility issues and, as a result, both compounds were excluded from further analysis.

**Table 2:** Small molecule inhibitors of NUDT5 combining aspects of TH1713 and TH1533

The 16 NUDT5 inhibitors that met the IC_50_ < 100 nM criterion were subsequently screened for NUDT5 engagement at 20 µM in HL-60 cell lysates. Relative NUDT5 levels in the presence of compounds compared to a 37 °C control are shown in Fig. 3b. Of the evaluated inhibitors, compounds **25** (TH5423, MG assay IC_50_ = 54 nM), **26** (TH5424, MG assay IC_50_ = 39 nM) and **28** (TH5427, MG assay IC_50_ = 29 nM) showed the most stabilization of NUDT5 in cell lysates. These compounds, as well as **4** (TH1659, MG assay IC_50_ = 20 nM but was unable to stabilize NUDT5 above the 50 % cutoff) were then analyzed for target engagement with intact cells. At 20 µM, TH5423, TH5424 and TH5427 all stabilized NUDT5 to nearly the same levels as observed in the 37 °C DMSO control (Fig. 3c), whereas TH1659 showed a smaller degree of stabilization. ITDRF_CETSA_ illustrated that TH5427 was the most potent stabilizer under the described conditions with an apparent EC_50_ of 2.1 µM (Fig. 3d). Accordingly, TH5427 had a positive, dose-dependent shift in the apparent *T*_agg_—the temperature at which 50 % of the target protein denatures and aggregates (TH5427 shifted NUDT5 *T*_agg_ up to ≈ 8 °C; Supplementary Fig. 6). TH5423 and TH5424 could also dose-dependently stabilize NUDT5, however much higher concentrations were required. TH1659 demonstrated poor-to-moderate stabilization of NUDT5 in lysates and intact cells and was also distinctly toxic under tested conditions, which cast further doubt on its potential as an adequately selective NUDT5-targeted probe (Supplementary Fig. 7).

### NUDT5 target engagement by DARTS

While thermal stabilization assays are valuable methods for assessing target engagement,the dependency on heating can alter protein folding and ligand binding thermodynamics ^31^, i.e., the observed stabilization may not be physiologically relevant. Because NUDT5 had very high thermal stability (Supplementary Fig. 4b, *T*_agg_ ≈ 76–80 °C; consistent with MS-CETSA proteomic profiling^32^), it was especially pertinent to verify that TH5427 could engage NUDT5 at physiological temperatures. Drug affinity responsive target stability (DARTS)^33^ is an orthogonal assay based on the principle of protein stabilization against protease degradation in a cell extract (graphical procedure in Supplementary Fig. 8a). When added to HL-60 cells in culture, TH5427 (at 0.74 and 2.2 µM) stabilized NUDT5 against exogenous protease degradation following cell lysis, indicating potent target engagement (Supplementary Fig. 8b). In conjunction with the CETSA experiments, the DARTS data demonstrates TH5427 engages NUDT5 at physiological temperatures, further supporting its use as a targeted NUDT5 inhibitor.

### Crystal structure with lead NUDT5 inhibitor TH5427

A crystal structure was obtained with TH5427 in the active site of NUDT5 (Fig. 3e, Supplementary Fig. 5b and Table 1). TH5427 bound in a similar fashion to TH1713, with the same primary coordinating interactions. The theophylline moiety is anchored in place by stacking interactions between Trp28 (chain A) and Trp46 (chain B) and two hydrogen bonds are observed between the theophyline carbonyl oxygen atoms and the amide nitrogen of Glu47 (chain B) and the guanidinyl group of Arg51 (chain A). As expected, the piperazine moiety extended out from the active site while the dichlorobenzene tail extended deep into a hydrophobic pocket in a static position. Overall, the orientations of TH1713 and TH5427 were very similar and the additional functionality at the tail and 8-positions did not cause drastic changes in the binding modality (Supplementary Fig. 5b).

### TH5427 selectivity

TH5427 was then screened *in vitro* against a panel of NUDIX enzymes and other nucleotide phosphohydrolases for potential off-target activity. Outside of NUDT5, TH5427 had the strongest activity against MTH1 (82 % inhibition) and gave 39, 66, and 38 % inhibition against dCTPase, NUDT12 and NUDT14, respectively, at 100 µM (Supplementary Fig. 1, Supplementary Fig. 9a). Of particular interest, TH5427 had no effect on NUDT9-mediated hydrolysis of ADPR, indicating discrimination amongst ADP-ribose hydrolases. Ensuing dose-response analysis for MTH1 gave an IC_50_ of 20 µM (*cf*. 29 nM for NUDT5), which results in an apparent 690-fold selectivity for NUDT5 over MTH1 in vitro (Supplementary Fig. 9b). Accordingly, 20 µM TH5427 treatment resulted in no thermal shift of MTH1 by CETSA (Supplementary Fig. 9c).

TH5427 selectivity was further scrutinized with the SafetyScreen 44 and ExpresSDiversity kinase panels from Eurofins Cerep Panlabs, which cover common off-target interactions typically seen during drug development and the diversity of the human kinome, respectively. Screened at 10 µM, TH5427 only displayed a few significant interactions within the chosen panels (including neurotransmitter transporters and hERG; Supplementary Fig. 9d and e). Altogether, these data suggest that TH5427 is a selective NUDT5 inhibitor with a promising safety profile.

### Exploring NUDT5 in breast cancer with targeted inhibitors

**Fig. 4:**
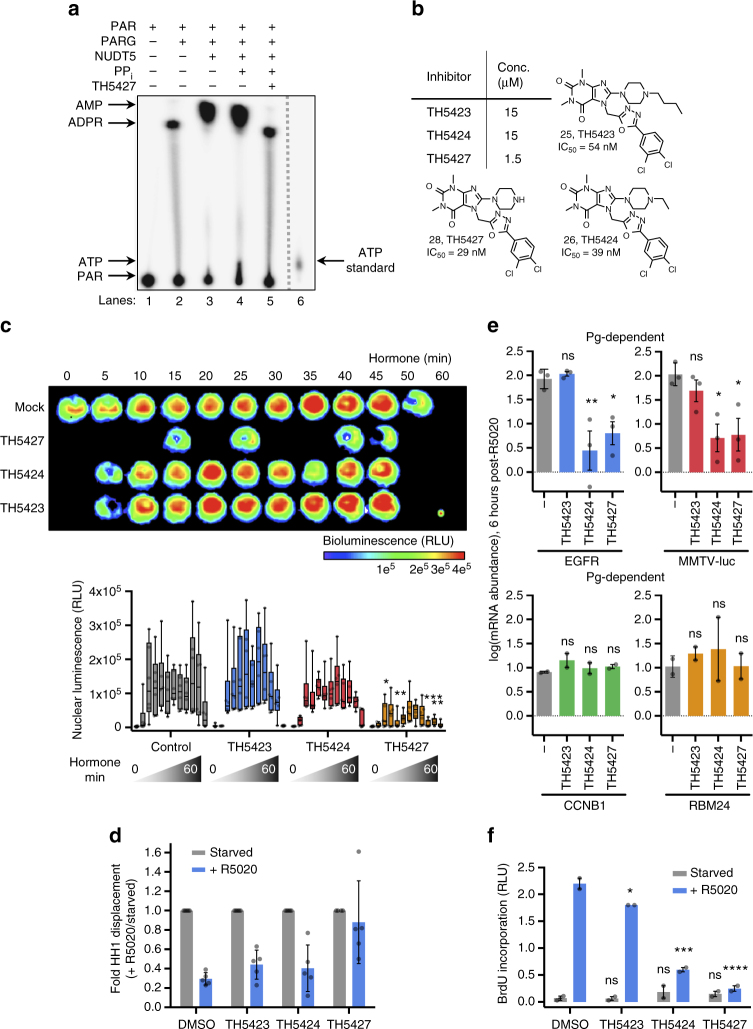
TH5427 inhibits progestin-dependent nuclear ATP synthesis in breast cancer cells. **a** Representative thin layer chromatography (TLC) of products formed following processing of ^32^P-PAR by recombinant PARG and NUDT5 in the absence or presence of TH5427 and PP_i_ in vitro (*n* = 2). **b** Concentrations and structures of inhibitors used in T47D^WT/M^ cell culture experiments. **c** Serum-starved T47D^WT^ cells expressing Nuc-luc FRTTO luciferase construct in the absence or presence of NUDT5 inhibitors prior to treatment with 10 nM R5020 and luminescence measurement. Top, a representative, pseudo-colored image of bioluminescence intensity over 60 min of R5020 treatment; bottom, box-and-whisker plots of bioluminescence quantitations from six independent experiments (center line, median; box limits, upper and lower quartiles; whiskers, minima and maxima; individual data points also shown). NS not significant, *p < 0.05, **p < 0.01, ***p < 0.001; repeated measures two-way ANOVA analysis. Control, gray, TH5423, blue, TH5424, red, TH5427, orange. **d** Histone displacement was determined by chromatin immunoprecipitation (ChIP) using a histone H1-specific antibody prior to (Starved, gray) or following 30 min of R5020 (+R5020, blue) in the presence or absence of NUDT5 inhibitors in T47D^M^ cells. Data from a representative experiment is presented as mean fold change (+R5020/Starved) ± SD of five different histone H1 contact regions. **e** Progesterone-dependent and –independent gene expression analysis in the presence or absence (−) of NUDT5 inhibitors following R5020 treatment (6 h) by RT-qPCR with T47D^M^ cells. Data represents the mean ± SEM log(mRNA abundance) normalized to serum-starved cells without R5020 treatment from three (progesterone-dependent) or two (progesterone-independent) independent experiments. *p < 0.05, **p < 0.01; one-way ANOVA analysis. **f** R5020-induced cell proliferation of T47D^M^ cells in the absence or presence of NUDT5 inhibitors was assayed by BrdU incorporation after 24 h. Mean ± SEM of individual BrdU chemiluminescence signals are displayed without R5020 treatment (Starved, gray) and following R5020 treatment (+R5020, blue) from two independent experiments. RLU; relative luminescence units. **p < 0.01, ****p < 0.0001; one-way ANOVA analysis

NUDT5 was recently identified as a key factor for the production of ATP in the nucleus of breast cancer cells, and its depletion blocked the regulation of progestin- and estrogen-dependent genes^23^. Notably, production of ATP from ^32^P-PAR was demonstrated using purified human NUDT5 and bovine PARG in vitro, which formed the basis of NUDT5′s involvement in hormone-dependent gene expression. Therefore, we first assessed the inhibitory effect of TH5427 in this same setting. When analyzed by thin layer chromatography (TLC), TH5427 inhibited the biochemical synthesis of ATP and AMP from ^32^P-PAR in the presence of purified NUDT5, PARG and PP_i_ (Fig. 4a, comparing lanes 4 and 5; Supplementary Fig. 1). This underscores a fundamental biochemical importance for NUDT5 activity in PAR-derived ATP synthesis.

To further demonstrate the utility of NUDT5-targeted probes, we analyzed our top compounds within the context of hormone signaling and breast cancer with T47D breast adenocarcinoma cells. For these experiments, lead NUDT5 inhibitor, TH5427, was used at 1.5 µM, whereas the less potent derivatives by CETSA, TH5423 and TH5424, were used at 10-fold higher concentrations (Fig. 4b). Similar to the target engagement experiments in HL-60 cells, TH5427 demonstrated the best ability to stabilize NUDT5 in T47D cells (Supplementary Fig. 10a). Likewise, TH5423, TH5424 and TH5427 were generally non-toxic when used below 20 µM (Supplementary Fig. 10b).

As NUDT5 activity is critical to elicit nuclear ATP production following hormone stimulation^23^, we used a nuclear-targeted luciferase reporter system to track nuclear ATP production after hormone treatment (Fig. 4c). Hormone-starved T47D^WT^ cells were incubated with NUDT5 inhibitors and then stimulated with the progesterone receptor agonist, R5020, for the time points indicated. While strong luminescence was observed in the cells treated with TH5423, TH5424 and the DMSO control, cells treated with TH5427 had a marked reduction in nuclear luminescence, indicating impaired nuclear ATP production (quantification Fig. 4c). This observation was consistent with previously reported siRNA experiments that showed NUDT5 depletion also attenuated nuclear ATP synthesis^23^. Additionally, the relative activity of the lead NUDT5 inhibitors was consistent with target engagement assays, where 1.5 µM of TH5427 had stronger effects than TH5423 or TH5424 at 15 µM.

Nuclear ATP feeds ATP-dependent chromatin remodeling enzymes, which disrupt the interactions of histones and DNA^23^. This process, in conjunction with histone acetyltransferase and deacetylase complexes, regulates the transcriptional activation of genes^34–36^. Blocking the production of nuclear ATP prevents the activation of ATP-dependent chromatin remodeling enzymes and displacement of histones H1 and H2A/H2B^23^. We therefore analyzed histone displacement following hormone exposure in T47D^WT^ cells treated with NUDT5 inhibitors (Fig. 4d). While moderate effects were seen with TH5423 and TH5424 (15 µM), treatment with TH5427 at 1.5 µM inhibited histone H1 displacement.

Without chromatin remodeling, the regulation of hormone-dependent genes and associated cell proliferation should also be impaired^23^. Using T47D cells harboring an integrated mouse mammary tumor virus luciferase reporter (MMTV-luc; T47D^M^ cells), mRNA expression for progestin-dependent genes (*EGFR* and *MMTV-luc*) was compared to progestin-independent genes (*CCNB1* and *RBM24*) following 6 h of R5020 treatment (Fig. 4e). Again, TH5427 significantly disrupted *EGFR* and *MMTV-luc* expression following progestin stimulation but did not affect the expression of *CCNB1* or *RBM24*. TH5424 showed similar activity to TH5427; however, TH5423 did not significantly disrupt progestin-dependent gene regulation.

Finally, as a consequence of blocking transcriptional responses, TH5427 treatment abrogated the progestin-dependent proliferation response in T47D^WT^ cells, as measured by BrdU incorporation (Fig. 4f). Collectively, these experiments confirm NUDT5 as an integral factor in progestin signaling and that TH5427 is a potent NUDT5 inhibitor that blocks hormone-dependent gene expression in breast cancer cells.

## Discussion

Investigating the role of NUDT5 in breast cancer has highlighted its importance in ADPR metabolism and hormone-dependent gene regulation. While early reports provided biochemical evidence that NUDT5 could hydrolyze 8-oxo-dGDP to 8-oxo-dGMP^14^, we were unable to demonstrate similar activity under physiologically relevant conditions. This was in line with reported suspicions that NUDT5 may have a limited cellular role in 8-oxo-guanine metabolism^13,37^. Furthermore, we have determined that NUDT5 depletion does not influence oxidized nucleotide content of DNA or increase DNA damage signaling under our treatment conditions, similar to previous studies with NUDT15 (MTH2)^38^. While this evidence suggests NUDT5 is not essential for 8-oxo-guanine sanitation, we cannot rule out situational contexts in which it may be important; as NUDT5 is clearly capable of hydrolyzing 8-oxo-dGDP *in vitro*^13,14,16,24,30,37^. On the other hand, we provide additional support that NUDT5 knockdown leads to impaired ADPR hydrolysis in cell lysates, which fits with previous reports pertaining to its role in ADPR metabolism^15,17,23^.

To further study NUDT5, we developed potent, small molecule inhibitors and probed their activity in cells with an emphasis on avoiding phenotypic biases by relying on thermal shift assays to evaluate inhibitor binding. Lead agent, TH5427, represents a potent NUDT5 inhibitor that was developed using a combination of target engagement techniques. This compound is non-toxic in cell lines tested up to 20 µM (Supplementary Figs. 7 and 10b) and does not have strong phenotypic effects when used in isolation. Similar to RNAi depletion, TH5427 neither induced significant DNA damage signaling nor increased OGG1-specific DNA lesions in cells when compared to the MTH1 inhibitor, TH1579 (Supplementary Fig. 11a–c). However, in combination with progestin stimulation, TH5427 blocked nuclear ATP synthesis, impaired hormone-induced chromatin remodeling, and disrupted progestin-dependent gene regulation and proliferation in T47D breast cancer cells. Its effective concentration was 10-fold lower than other synthesized NUDT5 inhibitors, consistent with our ranking based on target engagement assays.

In the development of effective probes or drug molecules, an important determinant of the quality and effectiveness relates to their ability to engage specific targets within the cell^39,40^. Recently developed and innovative target engagement assays (including CETSA and DARTS), have the potential to drive medicinal chemistry and drug discovery efforts, while also overcoming high attrition rates and lack of clinical efficacy with first-in-class drugs for validated targets^41,42^. This is reflected in recent paradigm shifts within the pharmaceutical industry, which have prioritized target engagement techniques to show drug efficacy in both pre-clinical and clinical settings^41^. In addition, the value of CETSA as a primary high-throughput screening assay was recently demonstrated for thymidylate synthase inhibitors^43^, further cementing cellular target engagement assays as powerful tools to identify lead molecules.

Utilizing these state-of-the-art techniques led us to discover TH5427, a potent and cell-active NUDT5 inhibitor that can be used to further understand the role of NUDT5 in biological systems. We have provided a proof of concept showing that TH5427 blocks NUDT5-dependent processes in breast cancer cells, and targeting NUDT5 may represent a promising new therapeutic approach for breast cancer treatment. Ongoing efforts are aimed at formulating NUDT5 inhibitors for in vivo use and will focus on further investigating the role of NUDT5 in cancer and other disease models.

## Methods

### Protein purification

Human NUDT5, NUDT12, NUDT14, NUDT15, and ITPase bacterial expression constructs in pNIC28 were a kind gift from SGC Stockholm. Human MTH1 (NUDT1) cDNA was codon optimized for *Escherichia coli* expression (Life Technologies) and subcloned into pET28a(+). Human NUDT9 cDNA was produced from HL-60 cells and subcloned into the pET28a(+) bacterial expression construct. All proteins contained N-terminal His-tags, were expressed in *E. coli* BL21 (DE3) R3 pRARE2 at 18 °C and purified by the Protein Science Facility (PSF) at the Karolinska Institute, Stockholm. Purification was performed using HisTrap HP (GE Healthcare) followed by gel filtration with a HiLoad 16/60 Superdex 75 (GE Healthcare)^4,9^. Human dCTPase and dUTPase were expressed as above but were purified using a HisTrap HP (GE Healthcare), the His-tag was removed by thrombin (dUTPase) or TEV digestion (dCTPase) and both were further purified by MonoQ ion exchange column^9^. Purity of the protein preparation was examined using SDS-PAGE followed by Commassie staining and the mass of the purified protein was determined using mass spectrometry. All purified hydrolases were concentrated, aliquoted and stored in storage buffer (20 mM HEPES, 300 mM NaCl, 10% glycerol, 1 mM TCEP, pH 7.5) at -80°C.

Purified GST-tagged human PARP1 and bovine PARG were produced with the pBC-PARP-1^44^ and pGEX-2T bPARG^45^ bacterial expression constructs, respectively^23^. Bacteria were cultured in 100 mL LB overnight at 37 °C and 180 RPM. Once the OD600 reading approached 1 the following day, expression was induced by the addition of IPTG (1 mM final concentration) and the cultures were grown for an additional 3 h. GST fusion proteins were then purified by affinity chromotography using a glutathione-Sepharose 4B resin and stored at -80 °C until use.

### In vitro analysis of substrate hydrolysis

Activity of NUDT5 and MTH1 with a panel of potential substrates [2-OH-ATP, 2-OH-dATP, 8-oxo-dGDP, GDP and 8-oxo-GTP (Jena Biosciences), 8-oxo-dGTP (TriLink Biotechnologies), dATP (ThermoFisher Scientific), dGTP, GTP and ADPR (Sigma-Aldrich)] was assessed in reaction buffer (100 mM Tris Acetate pH 7.5, 40 mM NaCl, 10 mM Mg Acetate) at 22 °C. For ADPR, alkaline phosphatase from bovine intestinal mucosa (10 U/ml; Sigma-Aldrich) was added as coupled enzyme, while other substrates were coupled to *E. coli* pyrophosphatase (0.2 U/mL, Sigma-Aldrich). After 30 min incubation with shaking, formed inorganic phosphate was detected by addition of malachite green reagent^27^. Absorbance at 630 nm was read after 15 min incubation with shaking using a Hidex Sense plate reader. The potency of NUDT5 inhibitors was assessed similarly as above (and in the screening campaign) by monitoring ADPR hydrolysis; details are in the Supplementary Information.

HPLC experiments were carried out with slight variations to previously described methods^5,46^. 50 µM ADPR or 8-oxo-dGDP were incubated with or without 6 nM NUDT5 in buffer containing 100 mM Tris-HCl, pH 7.4, 40 mM NaCl, 10 mM MgCl_2_, 1 mM dithiothreitol and 0.005 % Tween 20. Samples were incubated in 1.5 mL tubes in a water bath that was maintained at 37 °C and stirred using magnetic stir bars. After 30 min, 40 µL of the reaction mixture was added to 60 µL of cold MeOH (−20 °C) to quench enzymatic activity. The quenched samples were then cooled to −72 °C using an ethanol dry ice bath for several minutes. Samples were then clarified using a small bench-top centrifuge, and the supernatant (90 µL) was added to a fresh 1.5 mL tube. The water/methanol mixture was evaporated by vacuum centrifugation at 60 °C. Samples were then resuspended in ddH_2_O (90 µL), and transferred to a 96-well plate, which was then sealed and placed in the auto-sampler for HPLC analysis (samples were kept at 4 °C prior to injection).

The HPLC system was an Agilent 1100 equipped with an auto-sampler and a 5 µm C18 column, 4 × 125 mm column. Mobile phases consisted of a linear gradient of solvents from 40% A: 60 % B, to 100% B over 10 min then at 100% B for 2 min and a 3-minute equilibration back to 60 % B. Solvent A consisted of 10 mM KH_2_PO_4_, 10 mM tetrabutylammonium hydroxide, at pH 6.9 in H_2_O, and Solvent B consisted of 50 mM KH_2_PO_4_, 5.6 mM tetrabutylammonium hydroxide at pH 7.0 in H_2_O and 30 % methanol. Absorbance detection at 260 nm was used to observe the nucleotide species, retention times were verified using appropriate standards.

### Cell lines and culturing conditions

U-2 OS osteosarcoma and HL-60 acute promyelocytic leukemia cells were obtained from the American Type Culture Collection (ATCC, Manassass, VA, USA), and T47D breast adenocarcinoma cells (ER+/PR+/HER2-) were obtained from Karin Dahlman-Wright (Karolinska Institutet). U-2 OS cells were cultured in DMEM high glucose, GlutaMAX medium (Life Technologies/ThermoFisher Scientific), while HL-60 and T47D were cultured in RPMI, GlutaMAX medium (Life Technologies/ThermoFisher Scientific). All media were supplemented with 10% heat-inactivated fetal bovine serum (FBS) and penicillin/streptomycin. Unless otherwise indicated, cell cultures were maintained at 37 °C with 5% CO_2_ in a humidified incubator. The cells were routinely screened for mycoplasma using the MycoAlert kit (Lonza Bioscience) and none were listed on ICLAC or known to be cross-contaminated.

For hormone and inhibitor treatments, normal T47D^WT^ cells or cells containing a single copy of luciferase under the control of the mouse mammary tumor virus (MMTV) promoter (T47D^M^) were used^23^. Prior to hormone induction, cells were grown in phenol red-free RPMI medium supplemented with 10% dextran-coated charcoal-treated FBS (DCC/FBS). After 16 h in serum-free conditions, cells were pre-incubated with DMSO (vehicle) or NUDT5 inhibitor for 2 h at the indicated dose before addition of R5020 (10 nM) or vehicle (ethanol, EtOH) for the allotted time.

### Antibodies and reagents

Purified NUDT5 was submitted for polyclonal antibody production at EZBiolabs (USA). Anti-NUDT5 antibody was purified from rabbit serum on a home-made affinity column with immobilized NUDT5 using an AminoLink**™** Immobilization Kit (cat. no. 44890, Thermo Scientific). Anti-beta actin (β-actin, mouse monoclonal, cat. no. ab6276) and anti-53BP1 (rabbit polyclonal, cat. no. ab36823) were purchased from Abcam. Anti-γH2A.X (phospho-Ser139, mouse monoclonal, JBW301, cat. no. 05-636) was purchased from Millipore. Anti-SOD1 (rabbit polyclonal, FL-154, cat. no. sc-11407) was purchased from Santa Cruz Biotechnology. Anti-53BP1 (rabbit polyclonal, cat. no. A300-272) was purchased from Bethyl Laboratories. Anti-MTH1 (rabbit polyclonal, cat. no. NB100-109) was purchased from Novus Biologicals. Anti-RPA32 (rat monoclonal, cat. no. 2208S) and anti-Phospho-Histone H2A.X (Ser139; rabbit polyclonal, cat. no. 2577) were purchased from Cell Signaling. Donkey anti-mouse IgG IRDye 680RD (cat. no. 925–68072) and goat anti-rabbit IgG IRDye 800CW (cat. no. 925–32211) were purchased from Li-Cor. Goat anti-rabbit IgG Alexa Fluor 488 (cat. no. A-11008) and donkey anti-mouse IgG Alexa Fluor 647 (cat. no. A-31571) were purchased from ThermoFisher Scientific.

All NUDT5 inhibitors were dissolved in DMSO to a stock of 10 mM, and used at the final concentrations and incubation times indicated. MTH1 inhibitor, TH1579 (karonudib)^25^, was dissolved in DMSO and used at a final concentration of 500 nM and 24-hour incubation.

### siRNA transfections

The siRNAs used in these studies were purchased from Qiagen and are as follows:

AllStars Negative Control siRNA

siNUDT5 #1: 5′-CAAGAACCAACGGAATCTTCT-3′

siNUDT5 #7: Hs_NUDT5_7 Flexitube siRNA

siRNA transfection was performed using INTERFERin (Polyplus Transfection) at a final concentration of 10 nM and for 72 h prior to read-out, following the manufacturer’s instructions, unless otherwise noted.

### ADPR hydrolysis in cell lysates by HPLC

Cell lysates were generated to study NUDT5 enzymatic activities by HPLC based on earlier methods^26^. Briefly, 5 × 10^6^ U-2 OS cells per experimental condition were harvested by trypsinization and pelleted in microfuge tubes. siRNA was transfected for 72 h prior to analysis.

For analysis of cytosolic lysates, the cells were resuspended in 3–4 cell volume-equivalents of lysis buffer (20 mM HEPES, pH 7.4, 50 mM NaCl, 10 mM MgCl_2_, 1 mM EDTA, 5% (v/v) glycerol, 0.3 M sucrose, 0.3 M ammonium sulfate [(NH_4_)_2_SO_4_] and 1 mM dithiothreinol (DTT)) and incubated on ice for 15 min. The cells were then lysed with 10-15 strokes of a dounce homogenizer and nuclei/unlysed cells were pelleted by centrifugation for 10 min at 1000×*g* and 4 °C. The supernatants were transferred to new microfuge tubes and further clarified for 25 min at 16,000×*g* and 4 °C. The cytosolic supernatants were then saved for enzymatic analysis.

Lysates at cell concentrations of 5 × 10^7^ cells/mL were diluted 1:4 in malachite green assay buffer, pH 7.5, and 50 µM of substrate was added immediately before analysis. The enzymatic reactions were then conducted the same as with the in vitro studies for the indicated times. Enzymatic activity was then determined by HPLC analysis.

### Modified alkaline comet assay

The modified comet assay was used to determine relative amounts of OGG1-specific substrates (e.g., 8-oxo-dG) within cellular DNA^9^. U-2 OS cells were transfected with siRNA for 72 h and/or treated with DMSO/500 nM TH1579 for 24 h prior to harvesting by trypsinization. For experiments with TH5427, cells were treated for 24 or 72 h with 10 µM compound.

In brief, 120 µL of 1% agarose in PBS was added to one end of a fully-frosted microscope slide (ThermoFisher Scientific) and protected with a 24 × 40 mm coverslip to form the first layer. Once solidified at 4 °C, the second layer was made by mixing 400 µL of 1.2% low-melting agarose in PBS with 100 µL of a 1 × 10^6^ cells/mL suspension in PBS kept at 37 °C. The coverslip was removed and 80 µL was added on top of the first layer, spread with a coverslip, and left to solidify at 4 °C for 5–10 minutes. Two slides were made for each condition. The coverslips were again removed and the cells were lysed in lysis buffer (8.8 mM Tris-NaOH, pH 10, 2.2 M NaCl, 88.9 mM EDTA, 10% DMSO, and 1% Triton X-100) overnight at 4 °C in a microscopy slide staining jar, protected from light. The following morning, the slides were washed carefully with enzyme assay buffer (40 mM HEPES-KOH, pH 8.0, 0.1 M KCl, 0.5 mM EDTA and 0.2 mg/mL bovine serum albumin (BSA]) three times. The agarose was then covered with 100 µL of 0.8 µg/mL recombinant hOGG1 in enzyme assay buffer (or just assay buffer for controls), protected with a coverslip and incubated for 45 min at 37 °C. The slides were denatured in electrophoresis buffer (0.3N NaOH, 1 mM EDTA) for 30 min in a Comet Assay Tank (Thistle Scientific) at room temperature in the dark, followed by electrophoresis for 30 min at 25V and 300 mA. Finally, the slides were incubated for 45 min in neutralization buffer (0.4 M Tris-HCl, pH 7.5). Immediately before microscopy, 60 µL of SYBR Gold dye (Life Technologies, 1:1000 in PBS) was added to each sample.

The slides were visualized using a 10x objective and a Zeiss LSM 780 confocal or Zeiss Axiovert 35 microscope. Comets were analyzed with OpenComet (www.cometbio.org/) or Comet Assay IV (http://www.perceptive.co.uk/products/comet-assay-iv/) software and the tail moments (tail length x percent DNA in the tail) were automatically calculated. The tail moments were normalized to the buffer control for the siNon-targeting+DMSO or DMSO group to obtain relative tail moments. At least 100 cells were analyzed for each condition per replicate.

### Western blotting

Cells were lysed directly in 1× Laemmli buffer, boiled and sonicated. Proteins were separated by SDS-PAGE with 4–15% Mini-PROTEAN TGX gels and transferred to nitrocellulose with a Trans-Blot Turbo machine (Bio-Rad). Blocking of was performed with Li-Cor Blocking Buffer. Primary antibodies were incubated overnight at 4 °C in 1:1 Li-Cor Blocking Buffer/TBS + 0.05% Tween 20 (TBS-T) at the following concentrations: anti-NUDT5 (EZBiolabs + lab-purified, 1:1000), β-actin (Abcam, 1:5000), SOD1 (Santa Cruz, 1:1000) and MTH1 (Novus Biologicals, 1:1000). Secondary antibodies were diluted at 1:15 000 in 1:1 Li-Cor blocking buffer/TBS-T and incubated for 1 h at room temperature. Bands were visualized with an Odyssey Fc Imager and analyzed with Image Studio Software (Li-Cor Biosciences). All uncropped western blots from this manuscript are compiled in Supplementary Figure 12.

### Immunofluorescence

Following siRNA knockdown for 48 h, 10 000 U-2 OS cells were seeded per well in black, clear-bottomed 96-well plates (BD Falcon) and allowed to grow for an additional 48 h. For inhibitor treatment experiments, cells were plated at 2000 per well and 10 µM TH5427 was added the following day for an additional 72 h. Cells were washed with PBS and then fixed with 4% paraformaldehyde in PBS (Santa Cruz) with 0.5% Triton X-100 for 15 min. Primary antibodies were γH2A.X (Millipore, 1:1000 or Cell Signaling, 1:1000), 53BP1 (Bethyl, 1:1000 or Abcam, 1:500) and RPA32 (Cell Signaling, 1:1000) diluted in 3% BSA/PBS and incubated at 4 °C overnight. Alexa Fluor secondary antibodies (ThermoFisher) were used at 1:1000 diluted in 3% BSA/PBS for 1 h at room temperature. DNA was counterstained with DAPI and plates were visualized using an ImageXpress high-throughput microscope (Molecular Devices) with 20x objective. Image analysis was performed with CellProfiler software (Broad Institute).

### Small molecule screening campaign

Details regarding the small molecule screening campaign for NUDT5 inhibitors are described in the Supplementary Methods.

### Synthesis of inhibitors

NUDT5 inhibitors were synthesized according to the methods described in the Supplementary Methods.

### Target engagement analyses

Differential scanning fluorimetry (DSF)^5,47^ was performed with 5 µM purified NUDT5, 20 µM NUDT5 inhibitor and 5x Sypro Orange added per well added to a 96-well PCR plate. Each experiment was performed in duplicate. Temperature gradients from 25 to 95 °C (increasing at a rate of 1 °C per minute) were completed with readings every minute on a CFX96 Real-Time PCR machine (Bio-Rad). Melting temperatures were determined as the minimum of the negative first derivative plots with CFX Maestro software (Bio-Rad).

Target engagement in cells was determined by the cellular thermal shift assay (CETSA)^29^. Briefly, cell lysate thermal shift assays were performed with 1 × 10^6^ HL-60 cells per temperature/condition. Cells were collected and washed once with PBS and resuspended in TBS with protease inhibitor cocktail (Mini cOmplete, EDTA-free, Roche) at 60 µL/1 × 10^6^ cells. The cells were then aliquoted and lysed by freeze-thawing three times with 3-min incubations (3x, 3 min.+3 min.) using an ethanol/dry ice bath and water bath at 37 °C. The lysates were then centrifuged at 20,000×*g* for 20 min at 4 °C to remove cellular debris. Supernatants were then transferred to PCR strip tubes and treated with 0.5 µL DMSO (0.8% v/v final) or 20 µM inhibitor for 20 min at room temperature. Lysates with compounds were heated at the indicated temperature in a Veriti Thermal Cycler (Applied Biosystems) for 3 min, then cooled for another 3 min at room temperature. They were then centrifuged at 20 000 × *g* for 20 min at 4 °C to pellet protein aggregates and 45 µL was removed and prepared for western blotting analysis.

For CETSA experiments with cells treated in culture, 1 × 10^6^ HL-60 were treated with DMSO (0.1 % v/v final) or 20 µM compound for 3 h at 37 °C and 5% CO_2_ in a humidified incubator. T47D^WT^ cells were treated instead with 80 µM TH1659, 15 µM TH5423/5424 and 1.5 µM TH5427. The cells were harvested, washed twice with PBS and resuspended in TBS with protease inhibitors, as above. Heating was identical as before, except that immediately following heating, the cell suspensions were snap frozen in a dry ice/ethanol bath and freeze-thawed, as previously, to lyse cells. Centrifugation at 20 000 × *g* for 20 min at 4 °C removed cellular debris and protein aggregates, and lysates were prepared for western blotting.

Treatments, heating and lysis procedures for ITDRF_CETSA_ were performed identically to those for CETSA experiments, except NUDT5 inhibitors were added to cells at serial dilutions from 20 to 0.06 µM. Curves were fit by sigmoidal dose-response (variable slope) to derive EC_50_ values in GraphPad Prism 7.

To determine membrane integrity and cell viability at the temperatures used for CETSA experiments, HL-60 cells were heated identically to CETSA experiments and the cells were resuspended in 40 µg/mL propidium iodide (PI). The cells were analyzed on a Navios flow cytometer with Kaluza software (Becton Dickenson).

Target engagement was also determined by drug affinity responsive target stability (DARTS)^33^. HL-60 cells were treated in culture with DMSO (0.1% v/v) or TH5427 at 0.7 or 2.2 µM for 4 h. Following two PBS washes, the cells were lysed with M-PER extraction buffer (ThermoFisher) supplemented with protease inhibitor cocktail (Roche). The lysates were divided into 20 µg aliquots and followed by digestion with pronase at the indicated protein to pronase ratios for 30 min at room temperature. Samples were prepared for western blotting and NUDT5 stabilization was compared with SOD1 as a loading control.

### Cell viability assays

Cell viability was assessed by resazurin^9^. 5000 HL-60 cells (or 1000 U-2 OS/2000 T47D cells) were seeded per well on a 96-well plate. The following morning, compounds (or the equivalent amount of DMSO) were added to obtain the final concentrations indicated and the cells were incubated at 37 °C for 72 h with resazurin added at 0.02 mg/mL for the last 2–6 h. Resorufin fluorescence was detected at 600 nM with a Hidex Sense fluorescent plate reader. Viability curves were fit using the sigmoidal dose-response (variable slope) function in GraphPad Prism 7.

### Crystal structures of NUDT5 bound to ligands

The structure of NUDT5 in complex with inhibitors TH1713 and TH5427 was determined from single crystal x-ray diffraction of truncated NUDT5 with residues 1–210 (26 mg/ml in 0.1 M HEPES, pH 7.5, 300 mM NaCl, 10% glycerol, and 1 mM TCEP). Sitting drop vapor diffusion experiments were performed at 18 °C, and NUDT5 was mixed with reservoir solution (0.1 M Na Citrate pH 6.2, 0.15 M ammonium acetate, 33% PEG 3350) in a 1:1 ratio. Once crystals were formed dry inhibitor was added to the crystal drop and incubated for 24–48 h. Diffraction quality crystals were extracted and flash frozen in liquid nitrogen. Both data sets were collected at the 14.1 beamline at BESSY II (Berlin, Germany) at 100 K and 0.92 Å wavelength. Data reduction and processing were carried out using XDS^48^ and programs from the CCP4 suite^49^. Both structures were solved via molecular replacement with NUDT5 PDBID 3AC9. Initial models were built using Phenix AutoBuild program^50^ and CCP4 Arp/wARP^51^, followed by iterative building cycles using the Refine program in Phenix^52^. Both structures were solved in the C 1 2 1 space group with two molecules in the asymmetric unit forming the NUDT5 functional dimer. Supplementary Figure 5 contains an alternative view of the NUDT5 dimer bound with TH1713 (a), electron density maps for TH1713 and TH5427 bound in the active site (b) and stereo views of the TH1713 and TH5427 electron densities (c). Relevant crystallography statistics are shown in Table 1. The coordinates and structure factors for the structures presented in this paper were deposited in the PDB under code 5NQR (NUDT5_TH1713) and 5NWH (NUDT5_TH5427).

### Selectivity assays for TH5427

The selectivity assay for TH5427 against similar pyrophosphohydrolases was performed with MTH1, NUDT9, NUDT2, NUDT12, NUDT14, NUDT15, NUDT18, ITPase, dCTPase and dUTPase^9^. All analyses were performed by malachite green assay following 30 min incubation at room temperature in reaction buffer (100 mM Tris Acetate, pH 7.5, 40 mM NaCl, 10 mM MgAc, 1 mM DTT) with 100 µM TH5427 and preferred substrates for each enzyme (4.75 nM MTH1 with 100 µM dGTP, 8 nM NUDT15 with 100 µM dGTP, 8 nM NUDT2 with 16 µM AP4A, 2 nM NUDT5 with 50 µM ADPR, 20 nM NUDT9 with 50 µM ADPR, 20 nM NUDT12 with 50 µM NADH, 2 nM NUDT14 with 50 µM ADPR, 200 nM NUDT18 with 50 µM 8-oxo-dGTP, 0.2 nM ITPase with 25 µM, 35 nM dCTPase with 35 µM dCTP, and 1 nM dUTPase with 12.5 µM dUTP). MTH1, NUDT15, NUDT18, ITPase, dCTPase and dUTPase reactions were coupled with 0.2 U/mL pyrophosphatase, while NUDT2, NUDT5, NUDT9, NUDT12, and NUDT14 reactions were coupled by bovine intestinal phosphatase (10 U/mL).

TH5427 selectivity was also studied with the SafetyScreen44™ Panel and ExpresSDiversity Kinase Panel from Eurofins Cerep Panlabs at 10 µM, according to the company’s procedures.

### Preparation of ^32^P-PAR

^32^P-NAD-labeled PAR was prepared as follows: 5 µg of purified GST-PARP1 was incubated with 2 µg of activated calf thymus DNA and 10 mM NAD +/^32^P-NAD (Perkin-Elmer) at a ratio of 1:10 for 30 min at 25 °C and brought to a final volume of 100 µL with PARP reaction buffer (50 mM Tris, pH 7.5, 50 mM NaCl, 0.5 mM DTT, 0.1% Triton X-100 and protease inhibitors)^23^. After incubation, 0.4 µg of DNase was added to obtain DNA-free probes and the reaction was precipitated using TCA. The pellet was washed with acetone, dried and resuspended in 100 µl (10 mM Tris-HCl, pH 8.0, 1 mM EDTA, 0.1 % SDS, 100 µg/mL of Proteinase K) and incubated for 1 h at 37 °C, after which radio-labeled PAR was extracted using phenol/chloroform and ethanol precipitation. The pellet was resuspended in 50 µL (10 mM Tris-HCl, pH 8.0, 1 mM EDTA) and used for subsequent biochemical analyses.

### Biochemical conversion of ADPR to ATP by NUDT5

Enzymatic conversion of radiolabeled ^32^P-PAR by PARG and NUDT5 was performed using purified radio-labeled PAR (as described above) with the addition of recombinant bovine PARG in PAR glycohydroxylation buffer (PGB; 10 mM HEPES, pH 7.5, 150 mM KCl, 10 mM MgCl_2_)^23^. Recombinant PARG was added at the beginning of the reaction to a 10 µL final volume and incubated for 1 h at 25 °C. Recombinant NUDT5, pyrophosphate and/or TH5427 (0.6 µM) were added as indicated and incubated for an additional hour. Following termination of the reactions (95 °C for 2 min), products were visualized by spotting a fraction onto polyethyleneimine (PEI)-cellulose plates (Macherey-Nagel, Polygram CEL 300 PEI/UV254), and resolved in 0.3 M KH_2_PO_4_ at pH 3.5. Dried TLC plates were exposed on phosphoscreens and scanned using a Typhoon Trio (Amersham Biosciences).

### ATP visualization using bioluminescence imaging

T47D^WT^ cells were seeded in RPMI in black-walled 96-well plates and transfected using Lipofectamine 2000® (Invitrogen, manufacturer’s instructions) with the nuclear-targeted luciferase constructs (Nuc-luc FRTTO^53^) at a final concentration of 50 ng DNA/well for 48 h. 16 h prior to hormone (R5020, 10 nM) exposure, cells were synchronized by serum starvation. Bioluminescence was activated by addition of 100 µl D-luciferin (1 mg/mL water, Sigma) and ATP was quantified using an IVIS system (Xenogen Corp).

### Histone H1 displacement by chromatin immunoprecipitation

ChIP assays were performed using a histone H1-specific antibody, AE4 (Abcam)^54^. 1 × 10^7^ T47D^M^ cells (plated and treated with hormone and/or inhibitor, as described above) were cross-linked for 10 min with 1% formaldehyde. Lysates were sonicated (Biorupter sonicator, Diagenode) resulting in DNA fragments with a size range of 200-300 bp. Histone H1 was immunoprecipitated from 50 μg of chromatin and 40 μL of Protein A-Agarose Beads (Diagenode) in IP Buffer (100 mM NaCl, 50 mM Tris-HCl, pH 8.0, 5 mM EDTA and 0.5% SDS) with protease inhibitors (11836170001, Roche), and this was incubated for 16 h with end-over-end rotation at 4 °C. The beads were washed 3-fold with low salt buffer (140 mM NaCl, 50 mM HEPES, pH 7.4, 1% Triton X-100), twice with high salt buffer (500 mM NaCl, 50 mM HEPES, pH 7.4, 1% Triton X-100) and once with LiCl Buffer (10 mM Tris-HCl, pH 8.0, 250 mM LiCl, 1% NP-40, 1% sodium deoxycholic acid and 1 mM EDTA) and 1X TE buffer at 4 °C. Crosslinks were reversed at 65 °C overnight and the immunoprecipitated DNA was purified using phenol-chloroform and ethanol precipitation. The resultant eluted DNA was quantified by Qubit 3.0 Fluorometer (Life Technologies) and qPCR (LightCycler FastStart DNA Master SYBR Green I, Roche). The fold enrichment of target sequences in the immunoprecipitated (IP) compared to input (I) fractions were calculated using the comparative Ct method (2^Ct(IP)−Ct(I)^). Values are referred to as relative abundance over time zero (no R5020 treatment). Primer sequences are available on request.

### Progesterone-dependent gene expression by RT–qPCR

Total RNA and cDNA were prepared using an RNeasy Plus kit (Qiagen) and Superscript II kit (Invitrogen), respectively, according to the manufacturers’ instructions^54^. Quantification of mRNA abundance of specific gene products was performed by RT-qPCR (LightCycler FastStart DNA Master SYBR Green I), normalized to GAPDH and expressed as relative RNA abundance over time zero (no R5020 treatment). Primer sequences are available upon request.

### Proliferation analysis following R5020 treatment by BrdU

T47D^M^ cells (1 × 10^4^) were plated in a 96-well plate in the presence or absence of inhibitors 2 h prior to R5020 addition. The cell proliferation ELISA BrdU chemiluminescent assay (Roche) was performed according to the manufacturer’s instructions 24 h following hormone addition.

### Statistical analyses

Statistical analyses for relevant experiments were performed as follows with GraphPad Prism 7 software. The DNA damage markers in Fig. 1c were analyzed by Kruskal–Wallis test with Dunn’s correction for multiple comparisons (*n* = 3; H_γH2A.X_, 45.28, H_RPA_, 71.3, H_53BP1_, 5.061). Those in Supplementary Fig. 11a were analyzed by Mann-Whitney test, two-tailed (*n* = 2; U_γH2A.X_, 3086667, U_RPA_, 777581, U_53BP1_, 3714547). The alkaline comet assays were analyzed by Kruskal-Wallis test with Dunn’s correction for multiple comparisons and OGG1-treated samples were compared to DMSO or siNeg controls (*n* = 2 per set in duplicate; H_Fig1d_, 326.2, H_SFig11b_, 328.8, H_SFig11c_, 63.73). All analyses for CETSA experiments was performed by one-way ANOVA with Dunnett’s correction for multiple comparisons of *n* = 2 repeats (df_Fig3c_, 9, df_SFig4d_, 7, df_SFig10a_, 9; *F*_Fig3c_, 12.99, *F*_SFig4d_, 80.73, *F*_SFig10a_, 5.331). ITDRF_CETSA_ experiments for TH5427 were performed *n* = 3 times. CETSA melt curves and ITDRF_CETSA_ curves were fit by sigmoidal dose-response (variable slope) to derive EC_50_ values. Nuclear bioluminescence experiments were analyzed by repeated measures two-way ANOVA with Dunnett’s correction for multiple comparisons and represent *n* = 6 repeats (Total df, 287; *F*_interaction_, 2.385, *F*_time_, 18.38, *F*_treatment_, 3.474, *F*_subjects (matching)_, 12.56). mRNA expression was log-transformed and analyzed by one-way ANOVA with Dunnett’s correction for multiple comparisons (*n* = 3 for progesterone-dependent genes, *n* = 2 for progesterone-independent genes; df_EGFR_, 11, df_MMTV-luc_, 11, df_CCNB1_, 7, df_RBM24_, 7; *F*_EGFR_, 10.73, *F*_MMTV-luc_, 6.587, *F*_CCNB1_, 1.139, *F*_RBM24_, 0.2468). Cell proliferation experiments with BrdU labeling (*n* = 2) were analyzed by one-way ANOVA with Dunnett’s correction for multiple comparisons (df_Starved_, 7, df_+ R5020_, 7; *F*_Starved_, 0.7637, *F*_+ R5020_, 247.93). In all cases, NS not significant, *p < 0.05, **p < 0.01, ***p < 0.001 and ****p < 0.0001. With experiments analyzed post hoc for multiple comparisons, adjusted *p*-values are reported. Error bars represent SEM in all cases, except for substrate screening, ChIP and viability experiments, which indicate the SD and are intended to represent the variation among replicates/independent experiments.

### Data availability

Materials, protocols and other supporting data used in this study may be obtained from the corresponding authors upon reasonable request. The coordinates and structure factors for the structures presented in this paper were deposited in the PDB under code 5NQR (NUDT5_TH1713) and 5NWH (NUDT5_TH5427).

## Electronic supplementary material

Supplementary Information
