# Author Correction: Targeted NUDT5 inhibitors block hormone signaling in breast cancer cells

**DOI:** 10.1038/s41467-019-12806-1

**Published:** 2019-11-01

**Authors:** Brent D. G. Page, Nicholas C. K. Valerie, Roni H. G. Wright, Olov Wallner, Rebecka Isaksson, Megan Carter, Sean G. Rudd, Olga Loseva, Ann-Sofie Jemth, Ingrid Almlöf, Jofre Font-Mateu, Sabin Llona-Minguez, Pawel Baranczewski, Fredrik Jeppsson, Evert Homan, Helena Almqvist, Hanna Axelsson, Shruti Regmi, Anna-Lena Gustavsson, Thomas Lundbäck, Martin Scobie, Kia Strömberg, Pål Stenmark, Miguel Beato, Thomas Helleday

**Affiliations:** 1 Science for Life Laboratory, Division of Translational Medicine and Chemical Biology, Department of Medical Biochemistry and Biophysics, Karolinska Institutet, Solna, SE-171 21 Sweden; 2 Centre de Regulació Genòmica (CRG), Barcelona Institute for Science and Technology, Barcelona, E-09003 Spain; 3 Universitat Pompeu Fabra, Barcelona, E-08003 Spain; 4 Department of Biochemistry and Biophysics, Stockholm University, Stockholm, SE-106 91 Sweden; 5 Uppsala University Drug Optimization and Pharmaceutical Profiling Platform, Department of Pharmacy, Uppsala University, Uppsala, SE-751 23 Sweden; 6 Chemical Biology Consortium Sweden, Science for Life Laboratory, Division of Translational Medicine and Chemical Biology, Department of Medical Biochemistry and Biophysics, Karolinska Institutet, Solna, SE-171 21 Sweden

**Keywords:** Breast cancer, Screening, Target validation, Drug discovery and development

Correction to: *Nature Communications* 10.1038/s41467-017-02293-7, published online 17 January 2018.

The original version of this Article omitted the following from the Acknowledgements:

This work was funded by the European Research Council (695376) (T.H.).

This has now been corrected in both the PDF and HTML versions of the Article.

