# Supplementary material for: Targeted NUDT5 inhibitors block hormone signaling in breast cancer cells
Source: Nat Commun. 2018 Jan 17;9:250. doi: 10.1038/s41467-017-02293-7 (PMC5772648; doi:10.1038/s41467-017-02293-7)
Supplement: Supplementary file 1 — Supplementary Information [file 41467_2017_2293_MOESM1_ESM.pdf]

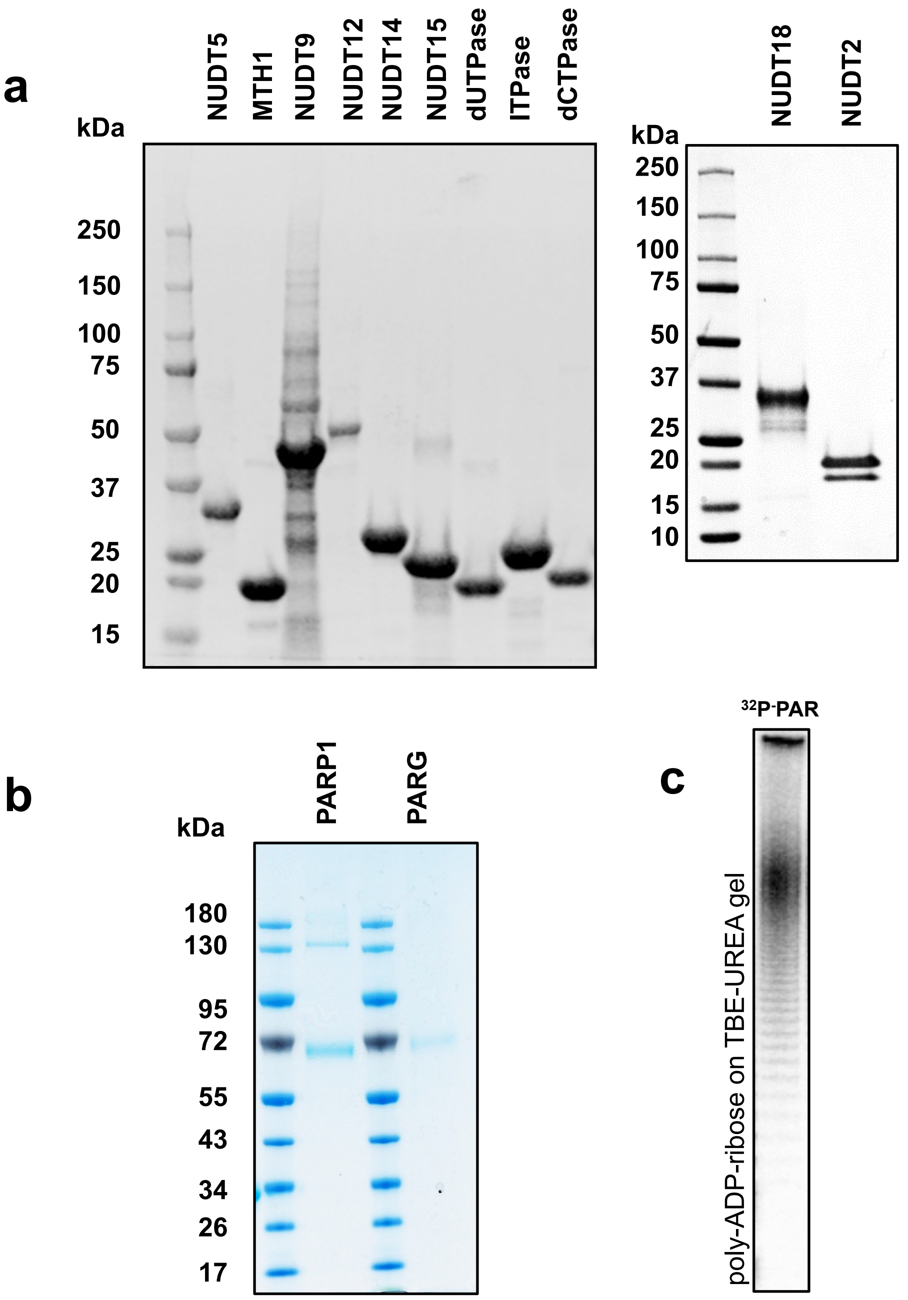

Supplementary Figure 1: All purified proteins and biopolymers used in this study. a, Purified human NUDT5, MTH1, NUDT9, NUDT12, NUDT14, NUDT15, dUTPase, ITPase, dCTPase, NUDT18 and NUDT2 were analyzed by SDS-PAGE and Coomassie staining. **b**, Purified human PARP1 and bovine PARG were analyzed by SDS-PAGE and Coomassie staining. **c**, ^{32}P-PAR from the reaction of ^{32}P-NAD with human PARP1 was analyzed by TBE-Urea gel electrophoresis.

Supplementary Table 1. Small molecule screening information

Category	Parameter	Description
Assay	Type of assay	*in vitro* target based
	Target	Human nudix hydrolase 5, NUDT5
	Primary measurement	Absorbance at 630 nm using a coupled enzymatic assay to detect inorganic phosphate (Pi) using the malachite green assay
	Key reagents	Human recombinant NUDT5 (In house produced in *E. coli*, see text for details)<br>ADP-Ribose (Sigma-Aldrich A0752)<br>Calf intestinal alkaline phosphatase CIP (Sigma-Aldrich P0014)<br>Malachite green detection reagents (Malachite Green, ammonium molybdate and Tween-20)
	Assay protocol	See supplementary information
	Additional comments	Protocol according to: Baykov, A. A., Evtushenko, O. A. & Avaeva, S. M. A malachite green procedure for orthophosphate determination and its use in alkaline phosphatase-based enzyme immunoassay. Anal. Biochem. 171, 266–270 (1988). Take care to ensure phosphate contamination in preparation of all reagents
Library	Library size	72055
	Library composition	The library consists of a chemically diverse collection of compounds containing both commercial (Enamine, TimTec, Maybridge and ChemDiv) and internal compounds (donation from Biovitrum). The library includes a small fraction of compounds with known bioactivities, *e.g.* the Prestwick set, and a set of nucleosides from Barry Associates.
	Source	The screen was done based on plating of 10 mM DMSO solutions from Labcyte 384 LDV plates using an Echo 550
	Additional comments	See below for further details on the composition of the Biovitrum derived compounds
Screen	Format	384-well format<br>Assay plate: 384-well PS plate, Nunc 242757
	Concentration(s) tested	Compound concentration at 10 µM, DMSO concentration at 0.1%
	Plate controls	Positive control: buffer only representing fully inhibited NUDT5 enzyme (16 on each plate)<br>Negative control: uninhibited NUDT5 enzyme (16 on each plate)
	Reagent/ compound dispensing system	Compound dispensing system: Echo 550 from Labcyte<br>Reagent dispensing system: FlexDrop IV from PerkinElmer<br>Multidrop from Thermo Scientific
	Detection instrument and software	Victor3 plate reader from PerkinElmer
	Assay validation/QC	Screen occasion 1: Positive control: average absorbance 0.24, standard deviation 0.01. Negative control: average absorbance 1.40, standard deviation 0.04. Average Z´ factor/plate: 0.87. Screen occasion 2: Positive control: average absorbance 0.24, standard deviation 0.02. Negative control: average absorbance 0.86, standard deviation 0.02. Average Z´ factor/plate: 0.85. QC also included monitoring of plate edge effects and hit distribution of the hits, with no corrections necessary
	Correction factors	Not applicable
	Normalization	Data are normalized to the positive (100% inhibition) and negative controls (0% inhibition) on each plate and are expressed as % inhibition
	Additional comments	The screen was performed at Chemical Biology Consortium Sweden at Karolinska Institutet, Sweden

Post-HTS analysis	Hit criteria	Screen occasion 1: Hit threshold = Average "% inhibition" of all test samples (-0,94%) + 3 times standard deviation of all test samples (3*3.7%) = 10.1%<br>Screen occasion 2: Hit threshold = Average "% inhibition" of all test samples (0,8%) + 3 times standard deviation of all test samples (3*5.9%) = 18.4%
	Hit rate	Screen 1: 0.3%<br>Screen 2: 0.8%
	Additional assay(s)	Retesting of hits in 3 concentration hit confirmation experiment followed by a full concentration response experiments at 11 concentrations
	Confirmation of hit purity and structure	ID and purity analysis with LC-UV/MS detection
	Additional comments	

Composition and storage of the Biovitrum derived compounds

Part of the CBCS screening library (appr. 8.000 compounds) was donated by Biovitrum AB in 2009. These compounds originated from both in-house and commercial sources. Compounds included in the screening set were selected to represent a diverse selection of a larger set of 65,000 compounds, while keeping a certain depth to allow crude structure–activity relationship studies. The selection was also biased towards lead-like and drug-like profiles with regards to molecular weight, hydrogen bond donors/acceptors and LogP[1].

Compound stock solutions at 10 mM in DMSO are stored frozen at approximately −20 °C in individual capped tubes in REMP 96 Storage Tube Racks. The racks are stored in a REMP Small-Size Store, which allows cherrypicking while the solutions are still frozen to minimize repetitive freeze-thaw cycles. For screening purposes the compound solutions have been replicated from the REMP racks to Labcyte 384 LDV plates (LP-0200) and then further into Labcyte 1536 HighBase plates (LP-03730) to enable dispensing using acoustic liquid handling equipment.

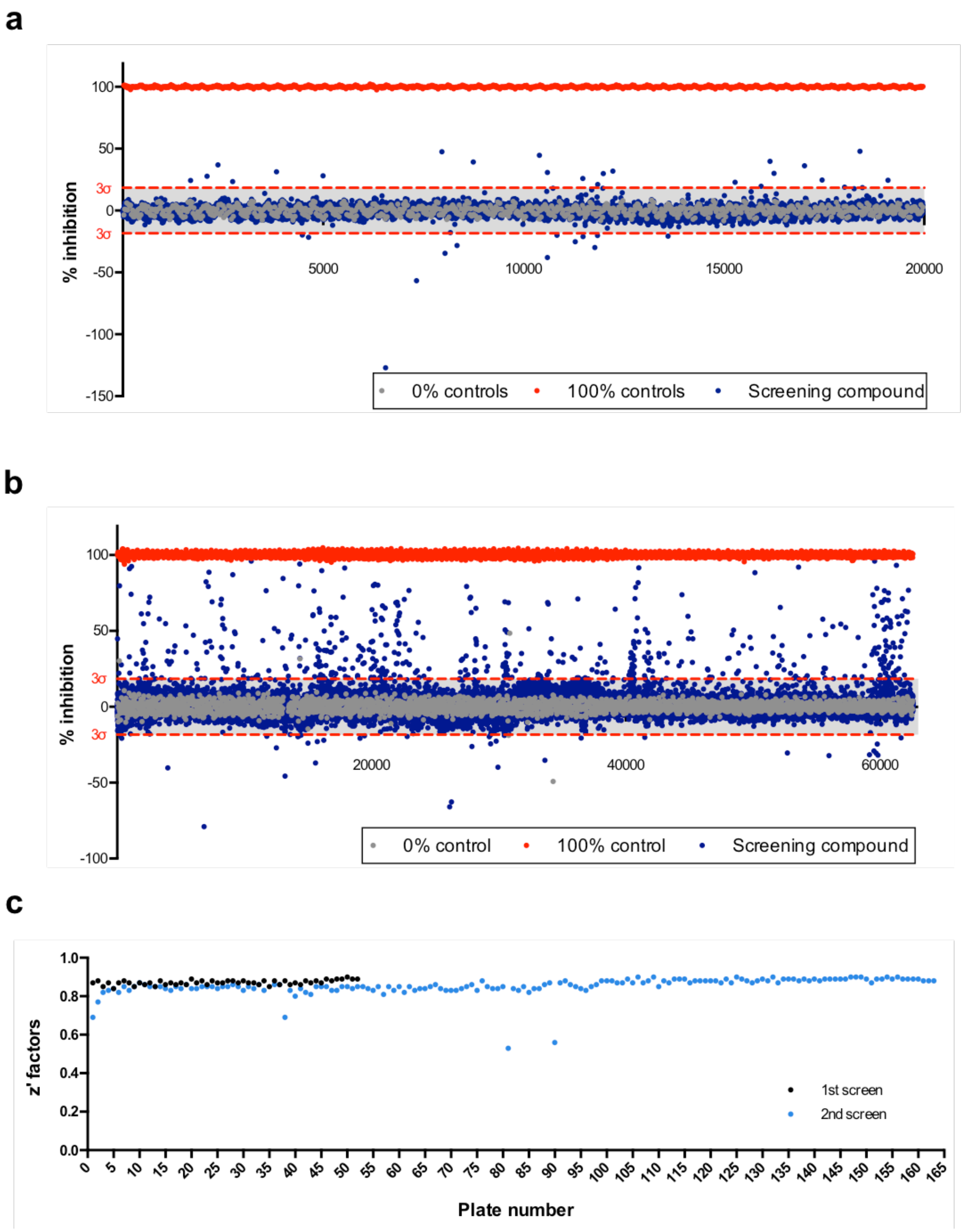

Supplementary Figure 2: Screening campaign for NUDT5 inhibitors. a, Scatter plot of hits from the first NUDT5 inhibitor screen using 40 mM NaCl, 10 mM Tris acetate and 10 mM Mg acetate. The hit limit (red, ±10.1) was defined as greater than the average of three standard deviations (3σ). **b**, Scatter plot of hits from the second NUDT5 inhibitor screen using 4 mM NaCl, 1 mM Tris acetate and 1 mM Mg acetate. The hit limit (red, ±18.4) was defined as greater than the average of three standard deviations (3σ). **c**, Plotted z' values for each plate of the high-throughput screen for NUDT5 inhibitors (black–1st screen; blue–2nd screen).

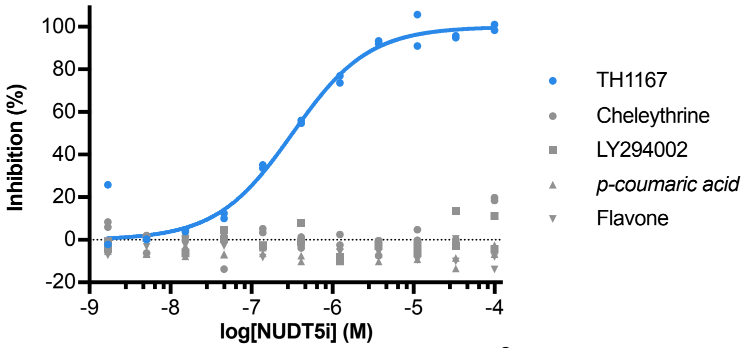

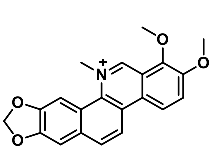

Chelerythrine

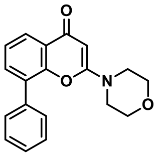

LY294002

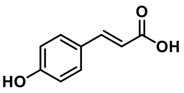

p-coumaric acid

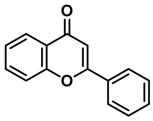

Flavone

Supplementary Figure 3: Previously proposed NUDT5/NUDT9 inhibitors compared with hit compound, TH1167, by ADPR hydrolysis via malachite green assay. Points represent n=2 experiments. The sigmoidal dose-response (variable slope) curve fitting from GraphPad Prism 7 was used for TH1167.

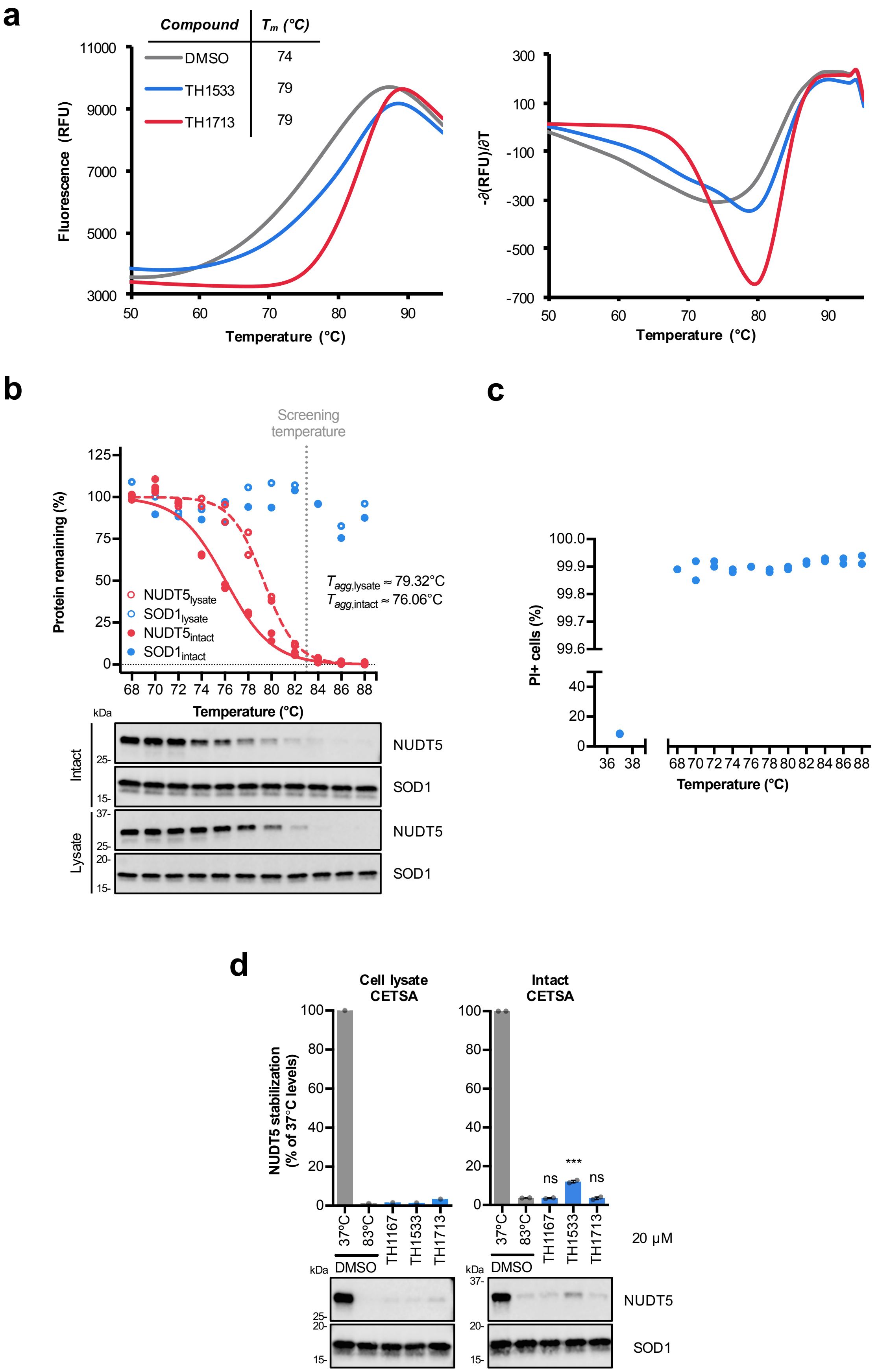

a
Compound
Tm (°C)
DMSO 74
TH1533 79
TH1713 79
Fluorescence (RFU)
Temperature (°C)
-∂(RFU)/∂T
Temperature (°C)
b
Screening temperature
Protein remaining (%)
NUDT5lysate
SOD1lysate
NUDT5intact
SOD1intact
Tagg,lysate ≈ 79.32°C
Tagg,intact ≈ 76.06°C
Temperature (°C)
kDa
Intact
Lysate
NUDT5
SOD1
NUDT5
SOD1
c
PI+ cells (%)
Temperature (°C)
d
Cell lysate CETSA
Intact CETSA
NUDT5 stabilization (% of 37°C levels)
ns
***
ns
37°C
83°C
TH1167
TH1533
TH1713
DMSO
20 µM
kDa
NUDT5
SOD1

Supplementary Figure 4: Developing a target engagement toolbox to study NUDT5 inhibition. **a**, Effects of TH1533 and TH1713 on NUDT5 stability by differential scanning fluorimetry (DSF). Left, raw fluorescence data (RFU, relative fluorescence units); right, melting temperatures were calculated from the minimum of the negative first derivative of fluorescence ($-\partial(RFU)/\partial T$) versus the temperature plot. A representative of two independent experiments is shown. **b**, NUDT5 aggregation temperature (T_{agg}) by CETSA melt curve with intact HL-60 cells and lysates. The thermally stable SOD1 was used as a loading control. Representative, cropped western blots are shown. Points represent n=2 experiments. **c**, Membrane integrity by propidium iodine (PI) permeation at temperatures used for NUDT5 CETSA analysis. Points are from n=2 experiments. **d**, Cell lysate and intact cell CETSA with initial NUDT5 inhibitors TH1167, TH1533 and TH1713. Cell lysate CETSA is a representative experiment (n=2) and intact cell CETSA is the mean of n=2 ± SEM. Individual data points are included and cropped, representative blots are displayed. NUDT5 stabilization is relative to NUDT5 band intensity at 37 °C and normalized to SOD1. ns – not significant; *** – $p<0.001$; one-way ANOVA.

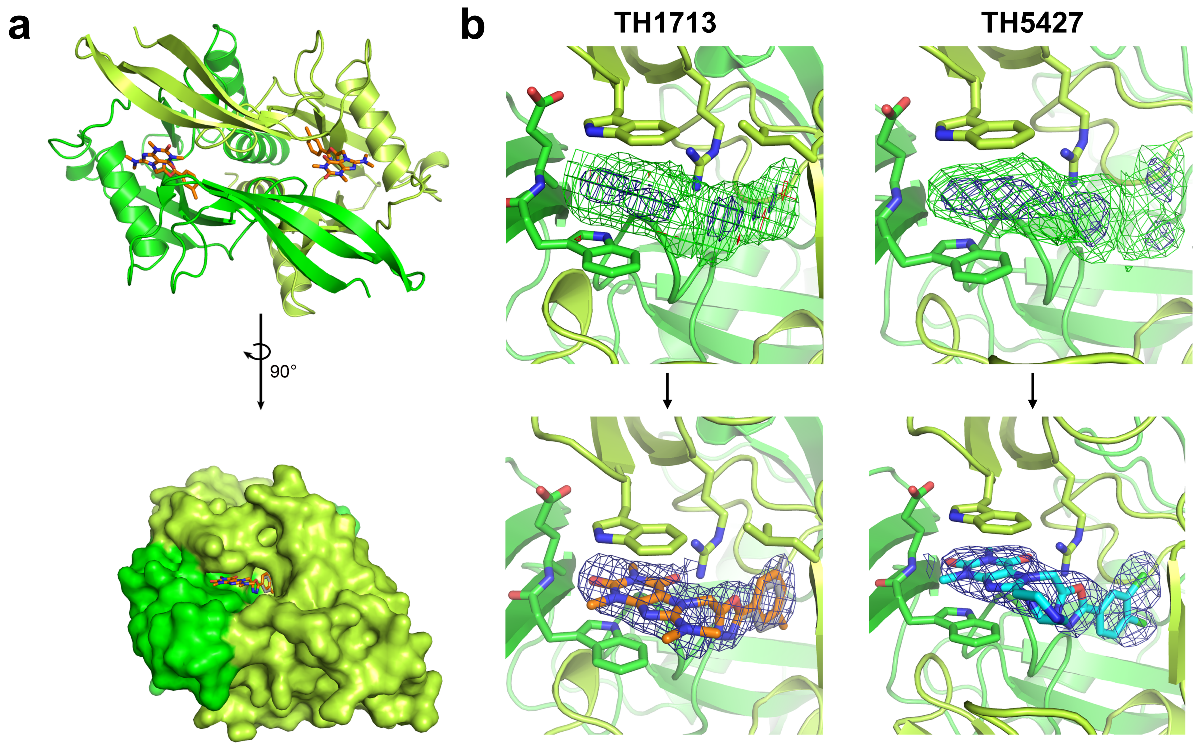
a
b
TH1713
TH5427
90°

c

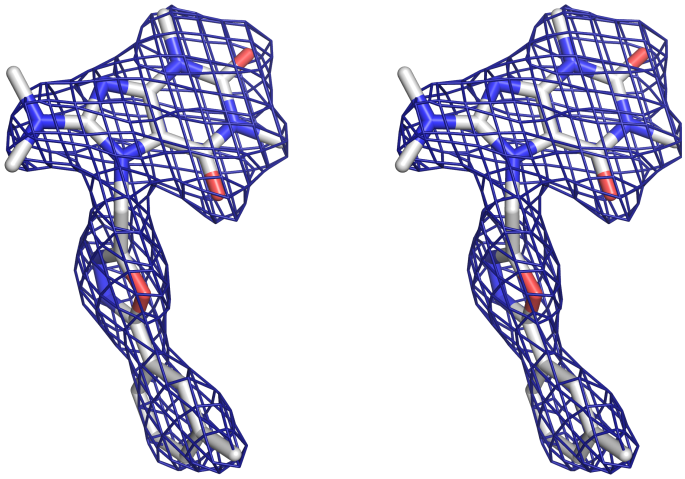

d

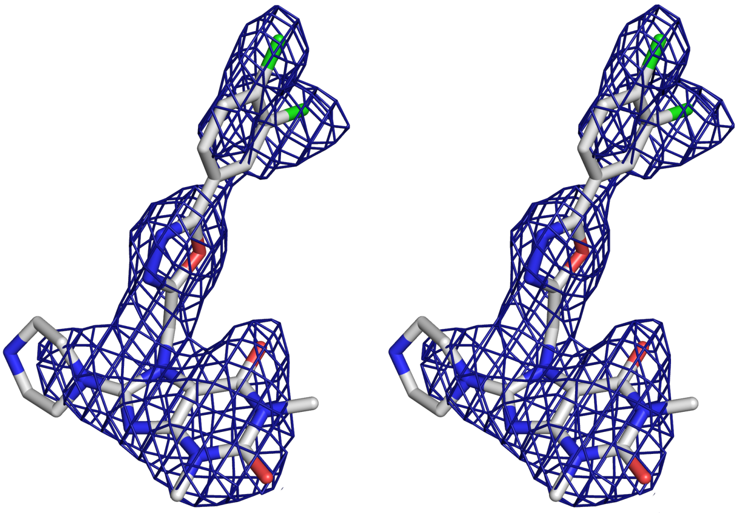

Supplementary Figure 5: Structure of NUDT5 dimer and different density maps for TH1713 and TH5427. a, NUDT5 dimer (chain A limon green and chain B green) with two molecules of TH1713 bound (orange) and a surface representation of the NUDT5 dimer oriented for viewing the binding pocket, which is comprised of residues from each monomer. **b**, Difference density omit maps for TH1713 (left) and TH5427 (right). Positive (green) and negative (red) difference density and $2F_O$-F_C density (blue) maps of the NUDT5 dimer (chain A limon green and chain B green) with inhibitors TH1713 and TH5427 omitted and 2.0σ level. Below, TH1713 and TH5427 in their final position with $2F_O$-F_C density map at 1.0σ (blue). **c**, Stereo view of TH1713 with its electron density ($2F_O$-F_C map contoured at 1.5σ; blue). **d**, Stereo view of TH5427 with its electron density ($2F_O$-F_C map contoured at 1.0σ; blue).

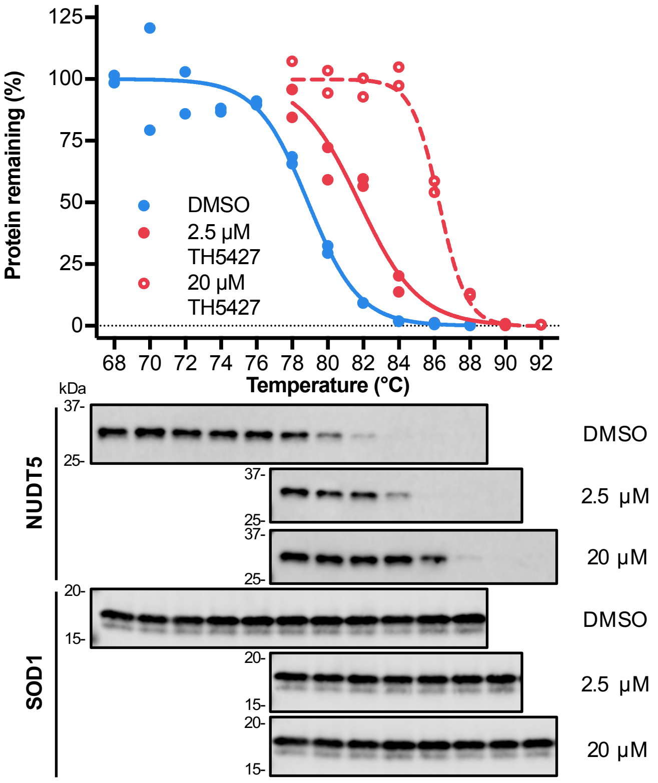

	DMSO	2.5 µM TH5427	20 µM TH5427
Apparent T_{agg}	78.93°C	81.98°C	86.27°C

Supplementary Figure 6: Thermal shift of NUDT5 with TH5427 by CETSA. CETSA melt curve comparing DMSO-treated HL-60 cells to those treated with 2.5 or 20 µM TH5427. All points are shown for n=2 experiments and are normalized to SOD1 band intensity as a loading control. Representative, cropped western blots are shown. Apparent T_{agg} temperatures are shown below.

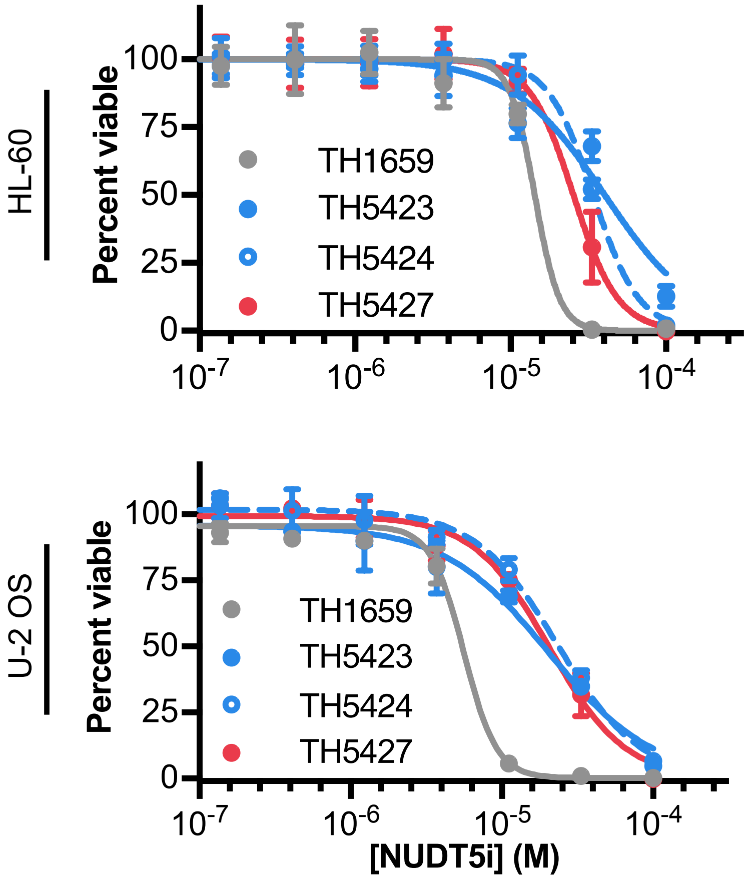

Supplementary Figure 7: The impact of NUDT5 inhibitors on cell viability. HL-60 or U-2 OS cells were treated with NUDT5 inhibitors for 72 hours and analyzed by resazurin assay. Points indicate the means ± SD of two independent experiments in duplicate.

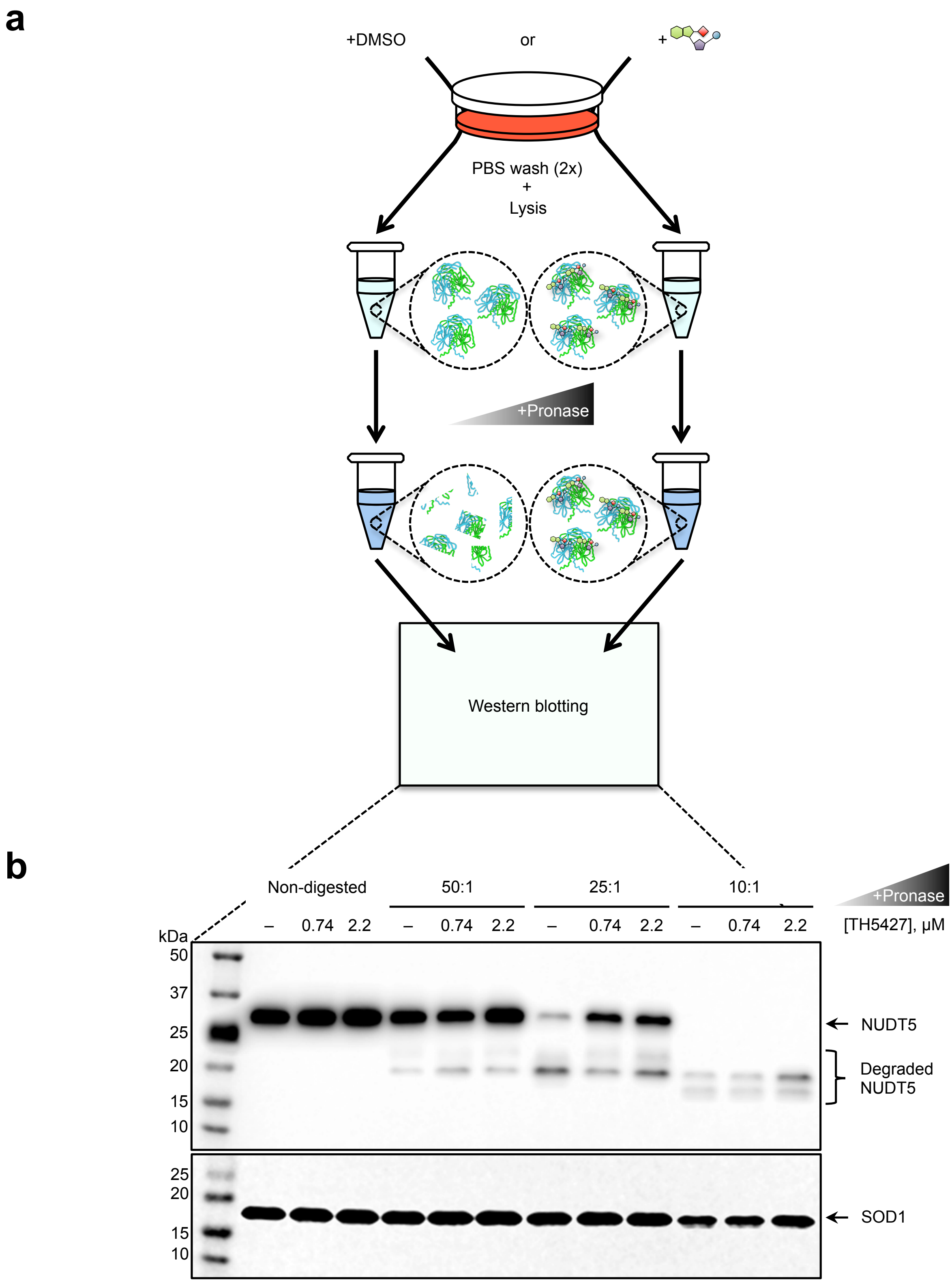

Supplementary Figure 8: Evaluation of TH5427 by DARTS. a, Schematic depicting the drug affinity responsive target stability (DARTS) assay. **b**, Stability of NUDT5 by DARTS assay following 4 hr treatment of HL-60 cells in culture with 0.74 or 2.2 μM TH5427. Protein lysate to pronase ratios are indicated and SOD1 was used as a loading control. A representative experiment is shown.

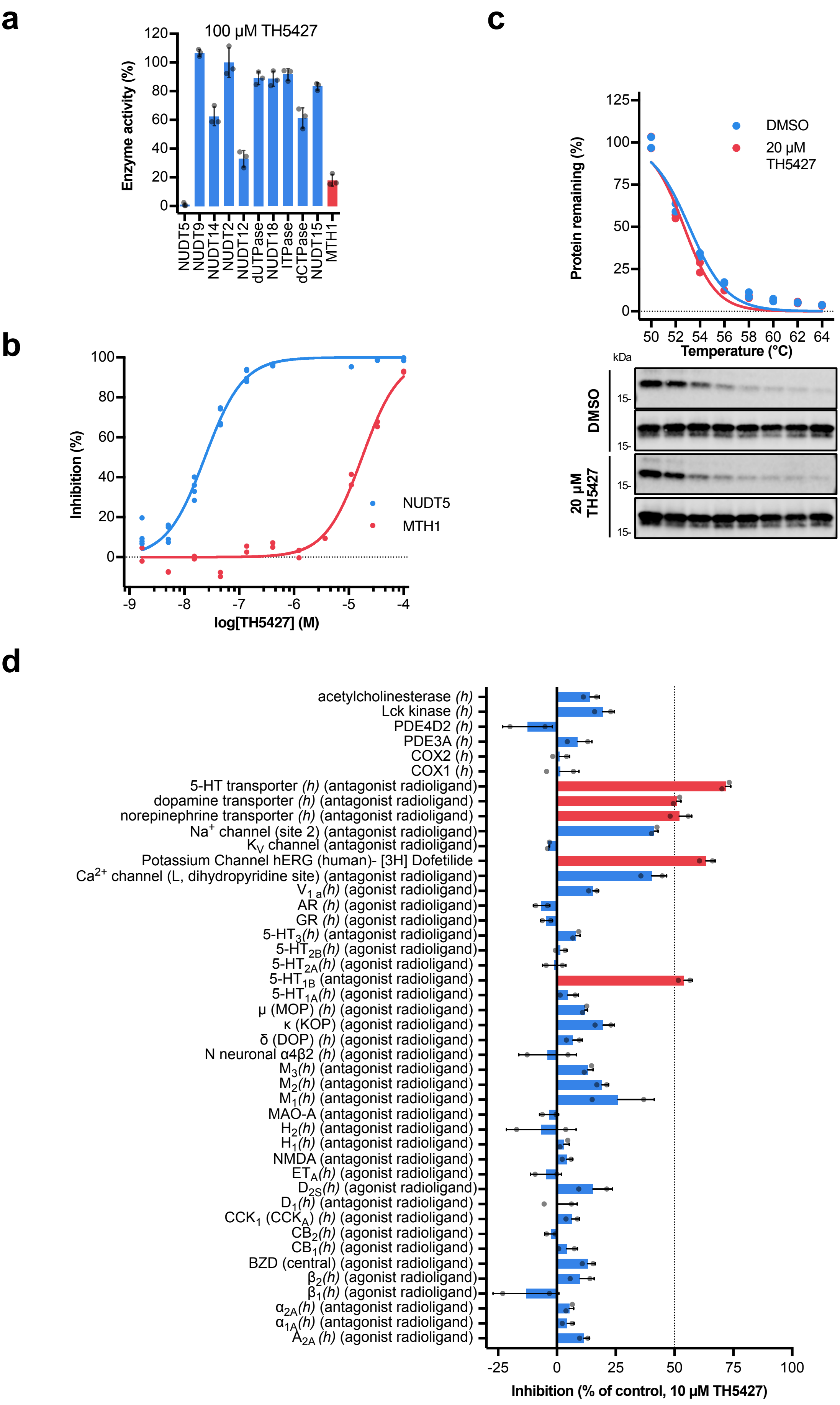
a
100 µM TH5427
Enzyme activity (%)
0
20
40
60
80
100
120
NUDT5
NUDT9
NUDT14
NUDT2
NUDT12
dUTPase
NUDT18
ITPase
dCTPase
NUDT15
MTH1
b
Inhibition (%)
0
20
40
60
80
100
NUDT5
MTH1
-9
-8
-7
-6
-5
-4
log[TH5427] (M)
c
Protein remaining (%)
0
25
50
75
100
125
DMSO
20 µM TH5427
50
52
54
56
58
60
62
64
Temperature (°C)
kDa
DMSO
20 µM TH5427
15-
MTH1
SOD1
d
acetylcholinesterase (h)
Lck kinase (h)
PDE4D2 (h)
PDE3A (h)
COX2 (h)
COX1 (h)
5-HT transporter (h) (antagonist radioligand)
dopamine transporter (h) (antagonist radioligand)
norepinephrine transporter (h) (antagonist radioligand)
Na^+ channel (site 2) (antagonist radioligand)
K_V channel (antagonist radioligand)
Potassium Channel hERG (human)- [3H] Dofetilide
Ca^{2+} channel (L, dihydropyridine site) (antagonist radioligand)
V_{1a}(h) (agonist radioligand)
AR (h) (agonist radioligand)
GR (h) (agonist radioligand)
$5\text{-}HT_3$(h) (antagonist radioligand)
$5\text{-}HT_{2B}$(h) (agonist radioligand)
$5\text{-}HT_{2A}$(h) (agonist radioligand)
$5\text{-}HT_{1B}$ (antagonist radioligand)
$5\text{-}HT_{1A}$(h) (agonist radioligand)
µ (MOP) (h) (agonist radioligand)
κ (KOP) (agonist radioligand)
δ (DOP) (h) (agonist radioligand)
N neuronal α4β2 (h) (agonist radioligand)
M_3(h) (antagonist radioligand)
M_2(h) (antagonist radioligand)
M_1(h) (antagonist radioligand)
MAO-A (antagonist radioligand)
H_2(h) (antagonist radioligand)
H_1(h) (antagonist radioligand)
NMDA (antagonist radioligand)
ET_A(h) (agonist radioligand)
D_{2S}(h) (agonist radioligand)
D_1(h) (antagonist radioligand)
CCK_1 (CCK_A) (h) (agonist radioligand)
CB_2(h) (agonist radioligand)
CB_1(h) (agonist radioligand)
BZD (central) (agonist radioligand)
$β_2$(h) (agonist radioligand)
$β_1$(h) (agonist radioligand)
$α_{2A}$(h) (antagonist radioligand)
$α_{1A}$(h) (antagonist radioligand)
A_{2A} (h) (agonist radioligand)
-25
0
25
50
75
100
Inhibition (% of control, 10 µM TH5427)

e

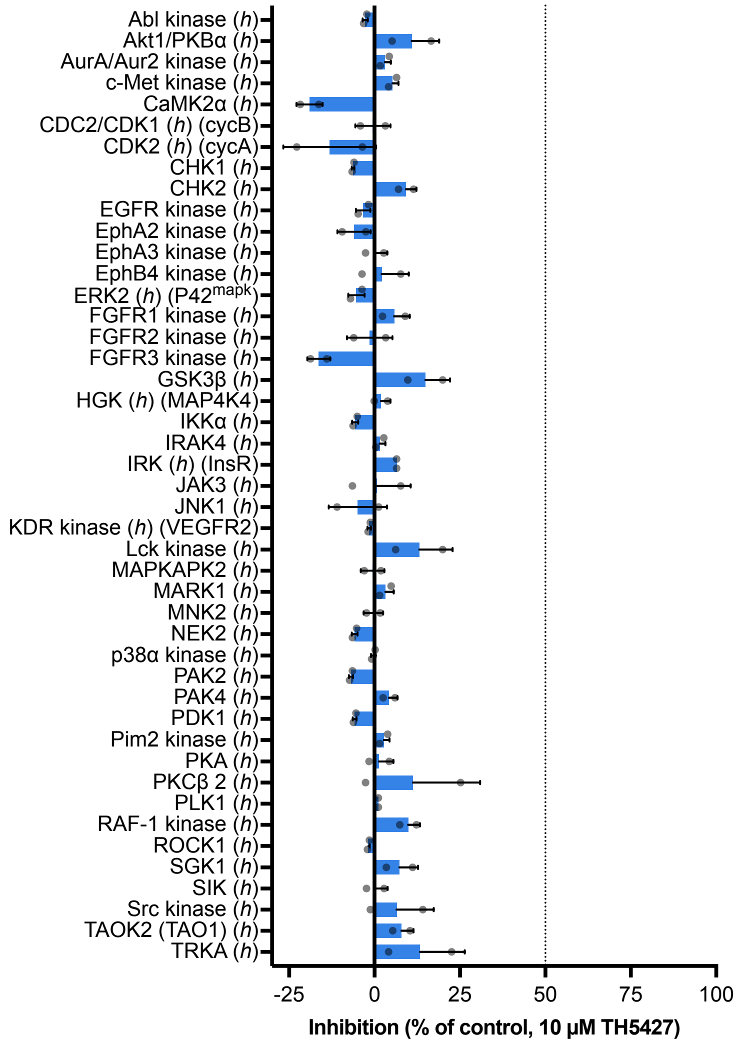

Supplementary Figure 9: Selectivity of TH5427. a, Selectivity against similar phosphohydrolases with TH5427 used at 100 µM by enzyme-coupled MG assay. Mean ± SD and individual values of a representative experiment are shown (n=2). **b**, Dose-response experiment in the malachite green assay comparing NUDT5 and MTH1 inhibition by TH5427. MTH1 IC_{50} = 20.3 µM *c.f.* NUDT5 IC_{50} = 29.1 nM. Individual points from two independent experiments (NUDT5) or a representative experiment (MTH1) in duplicate. **c**, CETSA melt curve for MTH1 after treatment with 20 µM TH5427. Individual points from two independent experiments, along with a cropped, representative western blot. **d**, TH5427 selectivity at 10 µM against the SafetyScreen44™ Panel from Eurofins Cerep Panlabs. The mean ± SD, as well as individual values, from two independent replicates are shown. Red bars indicate inhibition/activation of ≥ 50%. **e**, TH5427 selectivity at 10 µM against the ExpresSDiversity Kinase Panel from Eurofins Cerep Panlabs. The mean ± SD, as well as individual values, from two independent replicates are shown.

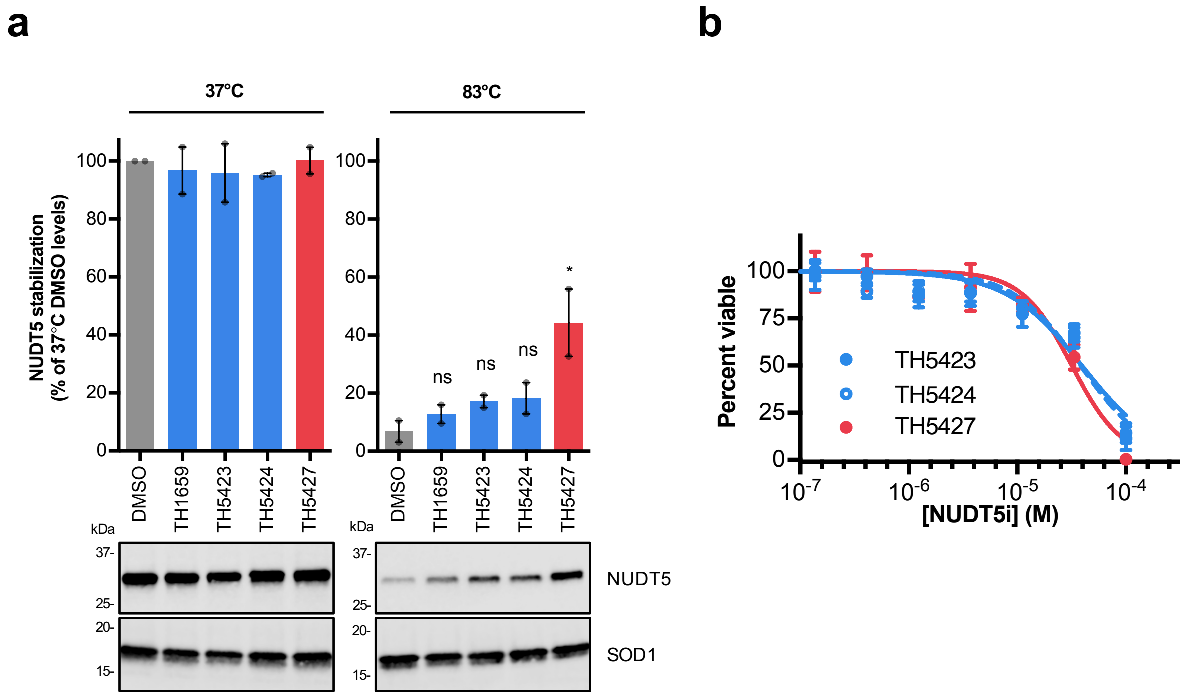

Supplementary Figure 10: NUDT5 inhibitor target engagement and effects on viability in T47D breast adenocarcinoma cells. a, NUDT5 CETSA with inhibitors at the specified concentrations. Points represent the means ± SEM from two independent experiments. Cropped, representative blots are provided. NUDT5 stabilization was relative to the 37°C DMSO control and SOD1 was used as a loading control. ns – not significant; * – p<0.05; one-way ANOVA. **b**, Viability by resazurin assay of T47D cells after 72 hours of NUDT5 inhibitor treatment. Mean values ± SD of n=2 experiments performed in duplicate.

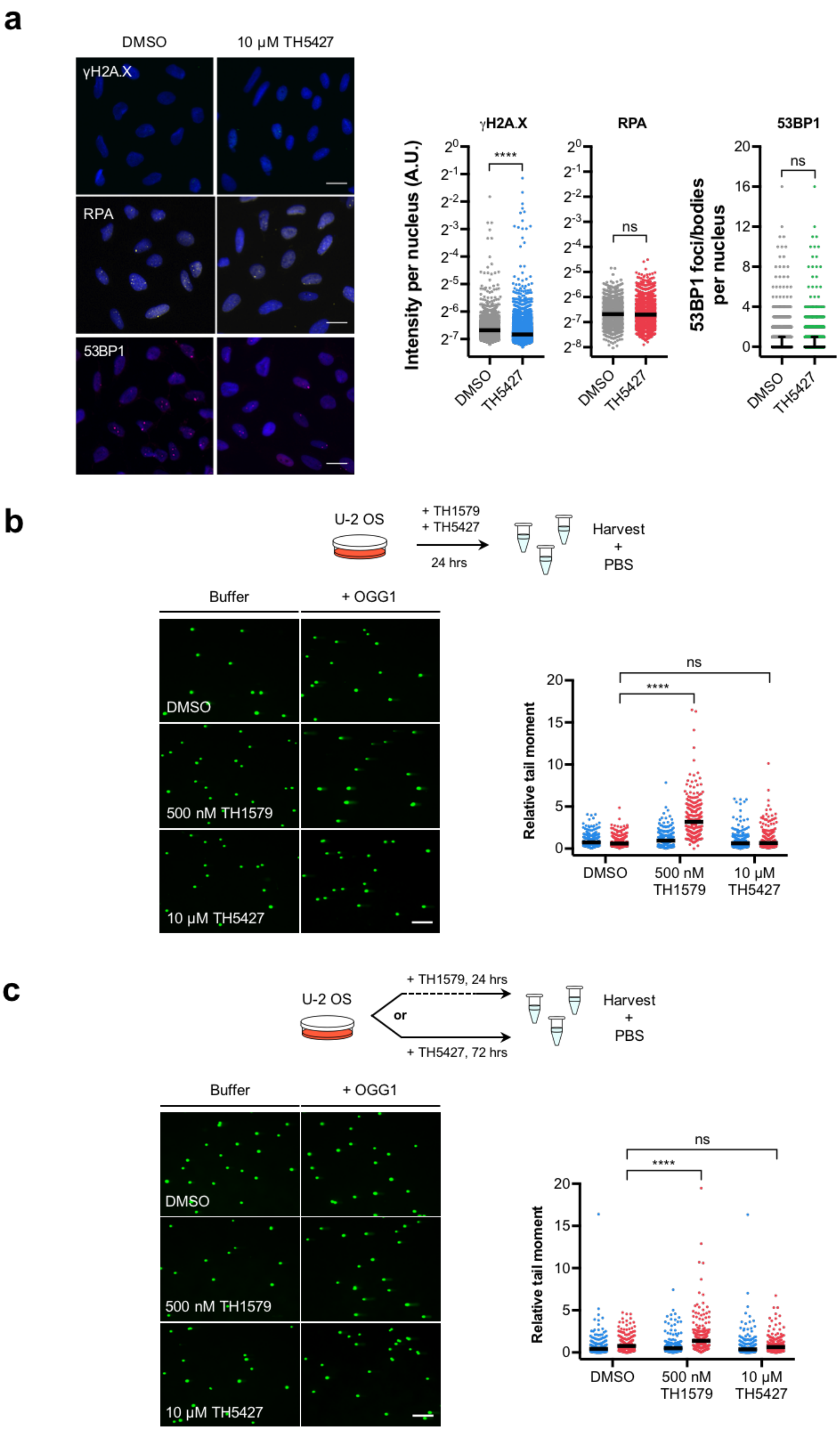
a
DMSO
10 μM TH5427
γH2A.X
RPA
53BP1
γH2A.X
RPA
53BP1
Intensity per nucleus (A.U.)
53BP1 foci/bodies per nucleus
****
ns
ns
DMSO
TH5427
b
U-2 OS
+ TH1579
+ TH5427
24 hrs
Harvest
+
PBS
Buffer
+ OGG1
DMSO
500 nM TH1579
10 μM TH5427
Relative tail moment
ns
****
DMSO
500 nM TH1579
10 μM TH5427
c
U-2 OS
+ TH1579, 24 hrs
or
+ TH5427, 72 hrs
Harvest
+
PBS
Buffer
+ OGG1
DMSO
500 nM TH1579
10 μM TH5427
Relative tail moment
ns
****
DMSO
500 nM TH1579
10 μM TH5427

Supplementary Figure 11: Probing the role of NUDT5 in 8-oxo-dG metabolism with TH5427. a, Left: Representative, pseudo-colored images of γH2A.X, 53BP1 and chromatin-bound RPA foci formation following treatment of U-2 OS cells with 500 nM TH1579 for 24 hours or 10 µM TH5427 for 72 hours. Scale bar = 20 µm. Right: Quantification of γH2A.X, 53BP1 and chromatin-bound RPA foci from two independent experiments (cells scored: γH2A.X – n=2959; RPA – n=1256; 53BP1 – n=2753). Lines for γH2A.X and RPA indicate median values; 53BP1, median values ± interquartile range. ns – not significant, **** – p < 0.0001; Mann-Whitney test, two-tailed. **b**, Left: Representative, pseudo-colored images of U-2 OS cells treated with 500 nM TH1579 or 10 µM TH5427 for 24 hours by modified alkaline comet assay. Right: Quantification of the tail moment (relative to DMSO, buffer-treated control) from a representative experiment (of n=2, with two replicate slides per experiment). Buffer-treated cells are shown in blue; OGG1-treated cells are shown in red. Lines represent the median tail moment. ns – not significant; **** – p < 0.0001; Kruskal-Wallis test. Scale bar = 200 µm. **c**, Left: Representative, pseudo-colored images of U-2 OS cells treated with 500 nM TH1579 for 24 hours or 10 µM TH5427 for 72 hours by modified alkaline comet assay. Right: Quantification of the tail moment (relative to DMSO, buffer-treated control) from a representative experiment (of n=2, with two replicate slides per experiment). Buffer-treated cells are shown in blue; OGG1-treated cells are shown in red. Lines represent the median tail moment. ns – not significant; **** – p < 0.0001; Kruskal-Wallis test. Scale bar = 200 µm.

Figure 1d

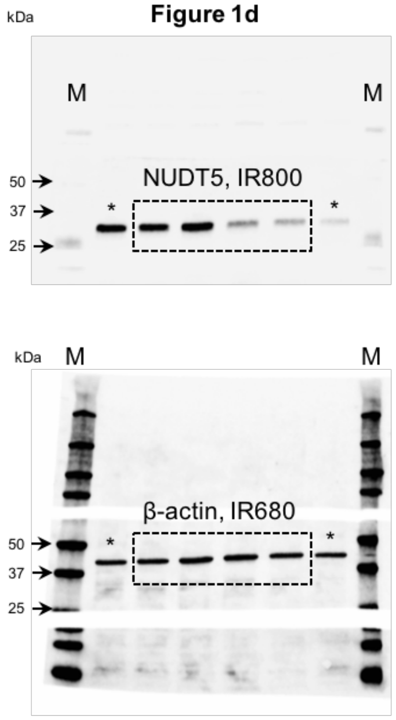

* – Transfection reagent vehicle control and additional NUDT5 siRNA not included in the current study

Figure 1f

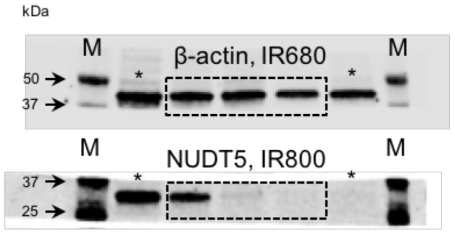

* – Transfection reagent vehicle control and additional NUDT5 siRNA not included in the current study

Figure 3b

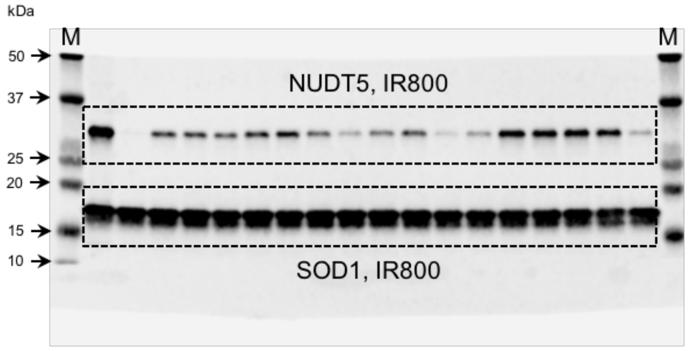

Figure 3c

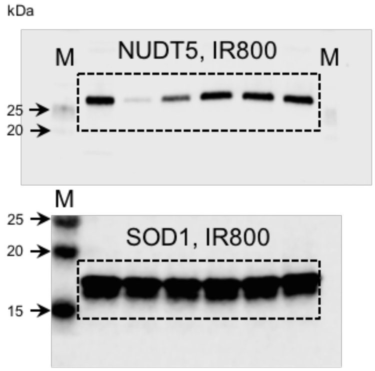

Figure 3d

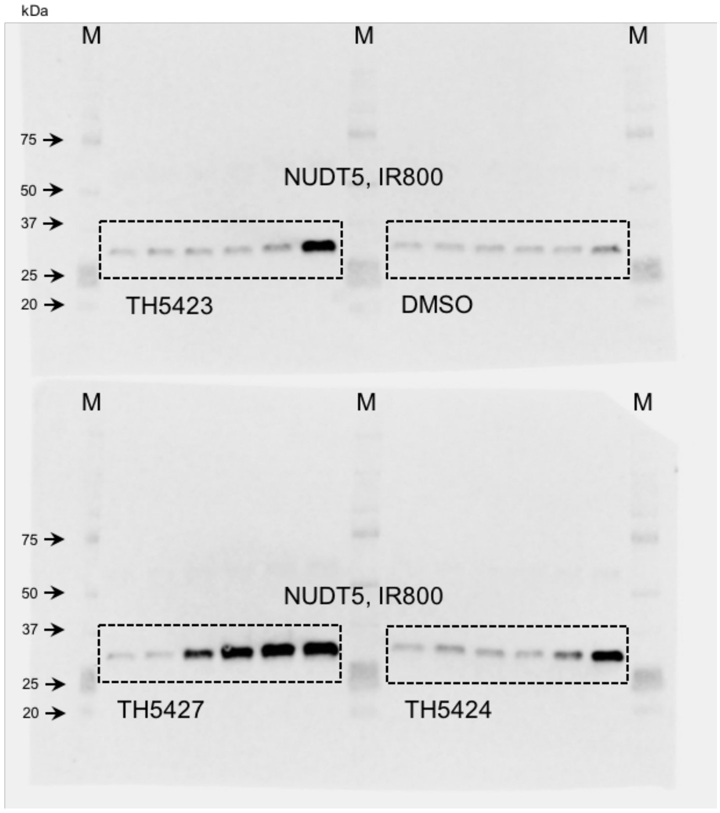

Supplementary Figure 9d

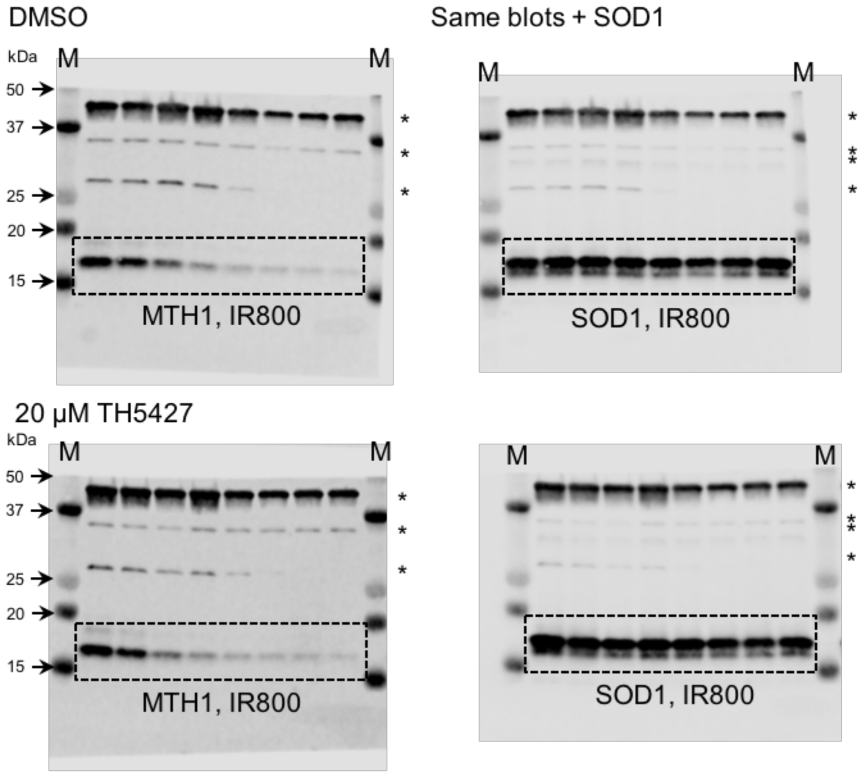

* – Non-specific bands from anti-MTH1 and/or anti-SOD1 antibodies.

Supplementary Figure 10a

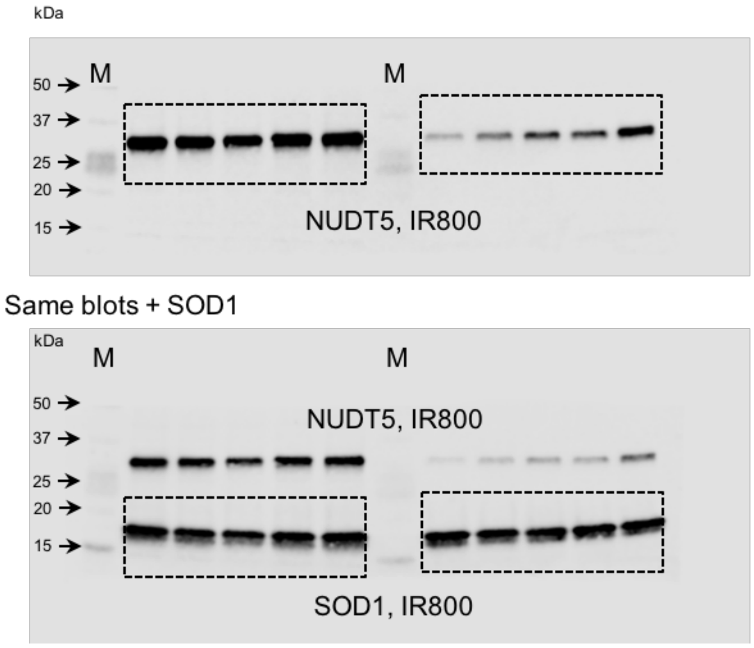

Supplementary Figure 4b

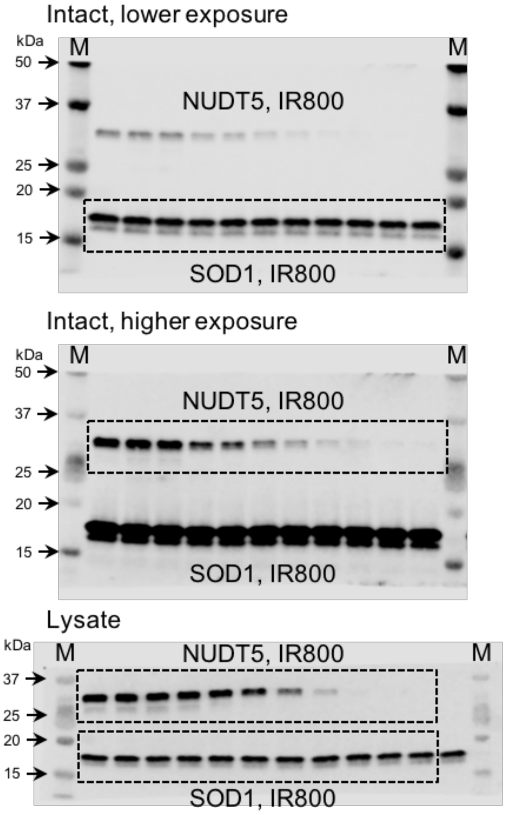

Supplementary Figure 4d

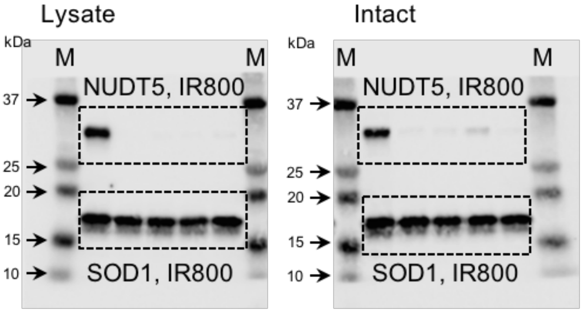

Supplementary Figure 5

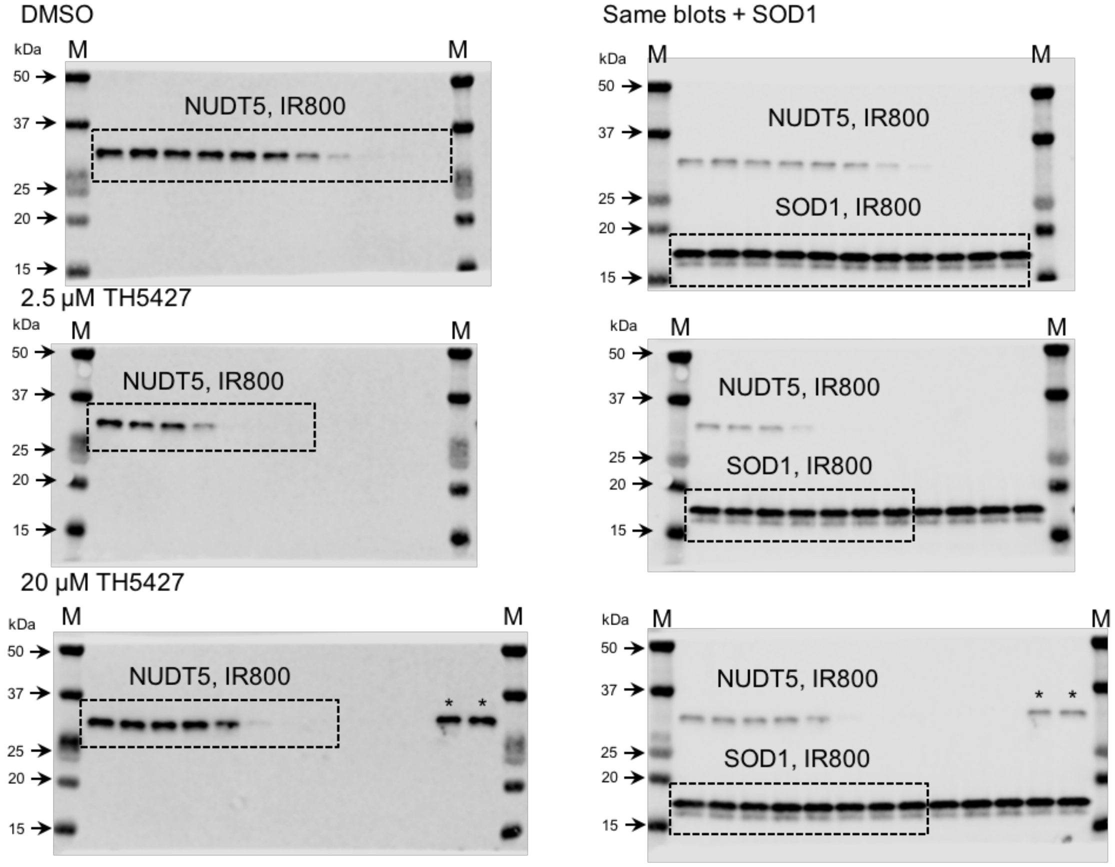

* – Misloaded samples from an adjacent, replicate blot. In instances where gradients reached the bottom plateau, blots were cropped at 90°C or 92°C.

Supplementary Figure 12: All uncropped western blots from other figures. Protein ladders are annotated as “M” for marker with molecular weight markers labeled, dotted lines indicate where cropping of blots occurred for figures and the primary/secondary antibodies used for each instance are labeled. Asterisks (*) denote irrelevant/erroneous samples or highlight non-specific bands recognized by the primary antibody.

Supplementary Methods

Small molecule library composition

The screen for NUDT5 inhibitors was conducted at Chemical Biology Consortium Sweden (www.cbcs.se). Two screening campaigns completed in August 2012 and in March 2013 respectively comprised a combination of in-house and commercially available libraries, amounting to a total of 75679 compounds. The commercial compounds originate from Enamine, TimTec, MayBridge and ChemDiv. The in-house libraries were donated by Biovitrum AB, Sweden (the origin and composition is described previously)[2]. For long-term storage, the compounds are kept frozen at -20°C as 10 mM solutions in dimethyl sulfoxide (DMSO) under low humidity conditions in REMP 96 Storage Tube Racks in a REMP Small-Size Store™. To facilitate screening an aliquot of the stock solutions have been transferred to Labcyte 384 LDV plates (LP-0200) to enable dispensing using an Echo 550™ acoustic liquid handler (LabCyte). For both screening campaigns 40 nl of the compound solutions were dispensed directly into columns 1-22 of the 384-well assay plates (Nunc 242757), while columns 23 and 24 were reserved for controls as outlined below. The plates were sealed with a Peelable Aluminium seal (Agilent 24210-001) using a PlateLoc thermal microplate sealer (Agilent) and kept at room temperature until used. The final compound concentration in the screen was 10 µM with a final DMSO concentration of 0.1% in all wells.

Enzymatic assay applied for screening and hit confirmation

The screen for inhibitors of NUDT5 was achieved using a coupled enzymatic assay with detection of inorganic phosphate (P_i) using the malachite green assay. Following enzymatic hydrolysis of ADP-Ribose (Sigma-Aldrich A0572) by NUDT5, to yield AMP

and Ribose-5-phosphate, the latter product is continuously processed by a significant excess of alkaline phosphatase (Sigma-Aldrich P4978 in first screen, P0114 in second screen) to ensure complete conversion. Formation of P_i is quantified based on the green complex (that absorbs at 630 nm) formed between malachite green and molybdate according to published procedure[3]. In the first screening campaign, the assay buffer consisted of 10 mM Tris-acetate at pH 8.0, 40 mM sodium chloride, 10 mM magnesium acetate, 0.005% Tween-20 and 1 mM dithiothreitol (DTT). The final assay during the enzymatic incubation was 1.5 nM recombinant human NUDT5, 50 µM ADP-Ribose and 5 U/ml of calf intestine alkaline phosphatase. In the second screening campaign, the assay buffer consisted of 1 mM Tris-acetate at pH 8.0, 4 mM sodium chloride, 1 mM magnesium acetate, 0.005% Tween-20 and 1 mM dithiothreitol (DTT). The final assay during the enzymatic incubation was 1 nM recombinant human NUDT5, 25 µM ADP-Ribose and 0.25 U/ml of bovine intestine alkaline phosphatase. In both screening campaigns the total assay volume was 40 µl, following the coupled addition of 10 µl of an enzyme solution and 30 µl of a substrate solution using a FlexDrop (PerkinElmer). In column 24, serving as a positive control and representing fully inhibited enzyme solution, the enzyme solution was replaced with buffer only. In column 23, serving as a negative control and representing uninhibited enzyme, had the same concentrations of enzyme and substrate with no compounds present. The microplates with compounds, enzymes and substrate were incubated at room temperature for 1h, after which the reaction was terminated and the signal developed by the addition of the malachite green reagent using a MultiDrop (Thermo Scientific). Following vigorous shaking on plate shakers for a minimum of eight minutes the absorbance was read in a microplate reader (Victor 3 from PerkinElmer) using a filter at 630 nm and a read time of 0.1 s per well. Raw data

from the Victor 3 instrument was imported into Microsoft Excel and normalized based on the negative and positive controls on each individual plate, such that the average response in column 23 of each plate defines 0% inhibition and the response in column 24 defines 100% inhibition. The same assay was applied also for hit confirmation purposes, although with 10, 40 and 80 nl dispensed to the 384-well assay plate to achieve a three-point concentration response at 2.5, 10 and 20 µM, respectively. Synthetic NUDT5 inhibitors were evaluated in the MG assay using the screening conditions from the first campaign. Inhibitors were used in dilutions from 100 µM to 1.7 nM in 3-fold dilution steps. Experiments were run in duplicate and confirmed in two independent experiments.

Chemical Methods:

Commercial reagents and solvents were purchased commercial suppliers and used without further purification. Essential reagents for this study include the following: *m*-toluyl chloride (99 %, Sigma Aldrich), 3,4-dichlorobenzoyl chloride (97 %, Sigma Aldrich), chloroacetic acid (99.5%, BDH Chemicals), $POCl_3$ (99 %, Sigma Aldrich), theophylline (≥ 99 %, Sigma Aldrich), 8-bromotheophylline (≥ 97 %, Fluka). Analytical thin layer chromatography was performed using 60-F-254 plates (E. Merck) and visualized using a UV lamp. Flash chromatography was performed with a Biotage SP4 MPLC system using Sigma Aldrich silica gel 60 Å (40−63 mm mesh size). ^{1}H NMR spectra were collected using a Bruker DRX-400 spectrometer. Chemical shifts of reported spectra are expressed in parts per million (ppm) and referenced to residual solvent peaks. Analytical HPLC-MS was performed using an Agilent MSD mass spectrometer connected to an Agilent 1100 system and a ACE 3 C8 (50 x 3.0 mm) column using a column temperature of 40 °C. Compounds were eluted in mobile phases A: H2O + 0.1 % TFA and B: MeCN in gradient from 10 to 90 % B over 1.5

minutes then increased to 99 % B (1 % A) over 0.1 minutes, and continued at 99 % B for 0.9 minutes. Total run time was 2.5 minutes at a flow rate of 1 mL/min. Retention times (t_R) are reported in minutes and all intermediates and final compounds were assessed to be >95% pure by HPLC-MS analysis, unless stated otherwise.

General Synthetic Protocols

General Procedure A: Synthesis of aromatic oxadiazoles:

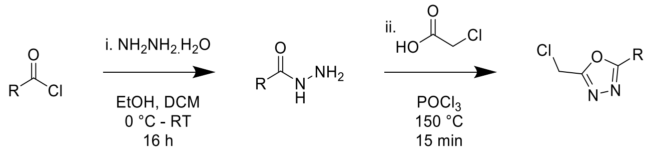

i) The corresponding acid chloride (1 equiv.) was dissolved in dichloromethane (0.2 mol/L) and then added dropwise to a stirring solution of hydrazine hydrate (4 equiv.) in EtOH (0.8 mol/L) at 0 °C. The reaction was allowed to warm to room temperature and monitored for completion using TLC. Upon completion, the dichloromethane was removed under reduced pressure. The resultant suspension was then cooled to 0 °C and the precipitated product was collected by suction filtration and washed with cold water. This crude product was dried overnight *in vacuo* and used without further purification.

ii.) Benzohydrazide (1 equiv.) was added to 2-chloroacetic acid (1 equiv.) dissolved in $POCl_3$ (0.1-0.5 mol/L). The vessel was thoroughly flushed with nitrogen gas and sealed, then the mixture was heated to 150 °C for 15 minutes in a Biotage microwave reactor. Solids were then rinsed down into the solution with a small amount of $POCl_3$ and the reaction was heated at 150 °C for an additional 5 minutes. Upon completion, $POCl_3$ was removed under reduced pressure. The crude mixture was purified using silica gel chromatography in a mixture of iso-hexane and ethyl acetate.

General Procedure B: Alkylation of theophylline derivatives.

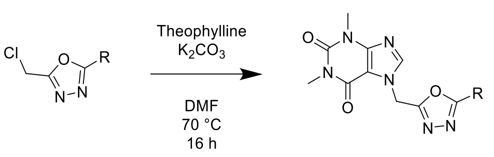

The corresponding 2-chloromethyl-oxadizole was added to a suspension of theophylline (or 8-functionalized-theophylline derivatives, 1.1 equiv.) and K_2CO_3 (1.2 equiv.) in DMF (~ 0.2 mol/L) and stirred at 70 °C overnight. Upon completion, the reaction mixture was diluted with H_2O and organics were extracted with EtOAc. The combined organics were washed with 1 M HCl, saturated $NaHCO_3$, H_2O and brine, followed by additional drying over $MgSO_4$. The crude mixture was then purified by automated flash chromatography in a mixture of iso-hexane and ethyl acetate.

General Procedure C: Aromatic substitution of 8-Br-theophylline

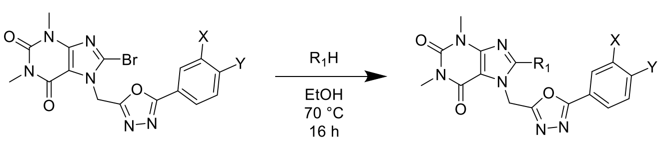

1. X = CH_3, Y= H
2. X = Y = Cl

Amines or thiols (2-5 equivalents) were added to functionalized 8-Br-theophylline suspended in ethanol, heated to 70 °C and stirred overnight. Upon completion, the reaction mixture was cooled to 0 °C and the precipitated product was collected by suction filtration. In cases where the product did not precipitate or was of insufficient purity, the crude mixture was purified using automated flash chromatography in mixtures of iso-hexane and ethyl acetate or dichloromethane and methanol. Compound **8** was prepared similarly to the above procedure; however, methanol was used as the solvent and sodium methoxide was used to displace the bromine.

General Procedure D: Synthesis of 8-alkyl-theophylline derivatives.

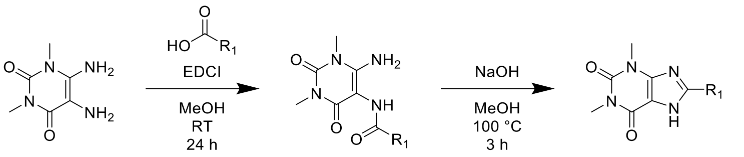

8-alkyl theophylline derivatives were prepared as previously reported.[4] Briefly, 5,6-diamino-1,3-dimethyl-1,2,3,4-tetrahydropyrimidine-2,4-dione was dissolved in methanol followed by the addition of the desired carboxylic acid and 1-ethyl-3-(3-dimethylaminopropyl)carbodiimide (EDCI). Upon disappearance of the starting material (~24 hours), methanol was evaporated and the crude mixture was purified by silica gel chromatography. The resultant carboxamide was then dissolved in a mixture of MeOH and 10 % NaOH in H_2O w/w (1:2) and stirred at 100 °C for 3 hours. Upon completion, the mixture was cooled to room temperature, acidified with HCl and the precipitated product was collected by suction filtration and washed with water.

Representative Syntheses:

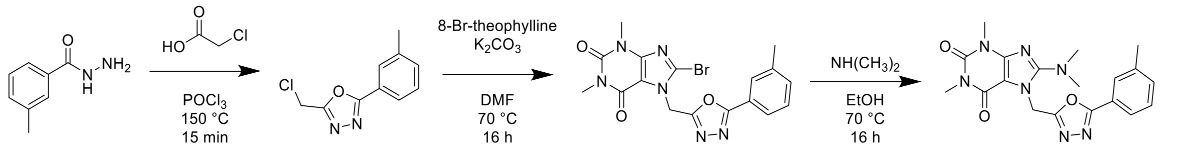

31 **32** **2**

Scheme 1. Synthesis of TH1713 (**2**).

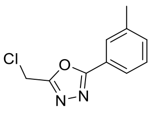

31. 2-(chloromethyl)-5-(3-methylphenyl)-1,3,4-oxadiazole was prepared as outlined in general procedure A on a 10 mmol scale. ^{1}H NMR (400 MHz, CHLOROFORM-*d*) δ ppm 2.45 (s, 3 H), 4.79 (s, 2 H), 7.30 - 7.47 (m, 2 H), 7.82 - 7.94 (m, 2 H). HPLC-MS t_R = 1.80 minutes, 90 % purity, m/z calculated for $[C_{10}H_9ClN_2O + H]$ = 209, found 209 (1.72 g, 83 %).

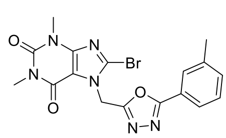

32. 8-bromo-1,3-dimethyl-7-{[5-(3-methylphenyl)-1,3,4-oxadiazol-2-yl]methyl}-2,3,6,7-tetrahydro-1H-purine-2,6-dione was prepared as outlined in general procedure B on a 0.9 mmol scale.

^{1}H NMR (400 MHz, CHLOROFORM-*d*) δ ppm 2.43 (s, 3 H), 3.41 (s, 3 H), 3.59 (s, 3 H), 5.90 (s, 2 H), 7.32 - 7.44 (m, 2 H), 7.73 - 7.82 (m, 1 H), 7.84 (dt, *J*=1.50, 0.83 Hz, 1 H). ^{13}C NMR (101 MHz, CHLOROFORM-*d*) δ ppm 21.3, 28.1, 30.0, 41.4, 108.9, 123.0, 124.2, 127.6, 128.3, 129.0, 133.0, 139.0, 148.3, 151.2, 154.2, 159.9, 166.1. HPLC-MS t_R = 1.70 minutes, 95.9 % purity, m/z calculated for [$C_{17}H_{15}BrN_6O_3$ + H] = 431.1, found 431.1 (199 mg, 55 %).

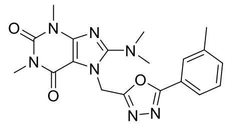

2, TH1713.

8-(dimethylamino)-1,3-dimethyl-7-{[5-(3-methylphenyl)-1,3,4-oxadiazol-2-yl]methyl}-2,3,6,7-tetrahydro-1H-purine-2,6-dione was prepared according to General Procedure C on a 0.06 mmol scale. ^{1}H NMR (400 MHz, CHLOROFORM-*d*) δ ppm 2.42 (s, 3 H), 2.98 - 3.11 (m, 6 H), 3.38 (s, 3 H), 3.55 (s, 3 H), 5.73 (s, 2 H), 7.30 - 7.46 (m, 2 H), 7.73 - 7.80 (m, 1 H), 7.80 - 7.86 (m, 1 H). ^{13}C NMR could not be obtained due to poor solubility. HPLC-MS t_R = 1.67 minutes, 100 % purity, m/z calculated for [$C_{19}H_{21}N_7O_3$ + H] = 396.2, found 396.2 (15 mg, 67 % yield).

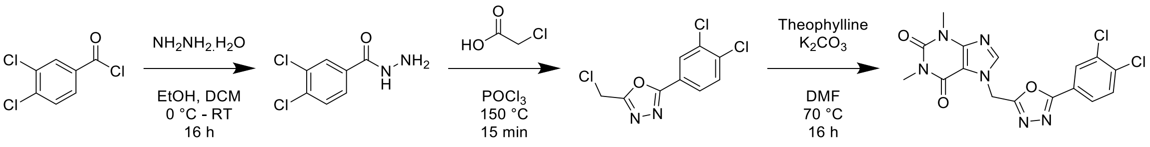

Scheme 2. Synthesis of TH1533 (**3**).

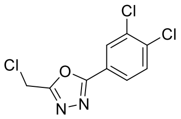

33. 2-(chloromethyl)-5-(3,4-dichlorophenyl)-1,3,4-oxadiazole was prepared according to General Procedure A on a 5 mmol scale. ^{1}H NMR (400 MHz, CHLOROFORM-*d*) δ ppm 4.79 (s, 2 H), 7.63 (d, *J*=8.37 Hz, 1 H), 7.93 (dd, *J*=8.37, 2.05 Hz, 1 H), 8.18 (d, *J*=2.05 Hz, 1 H). ^{13}C NMR (101 MHz, CHLOROFORM-d) δ ppm 32.9, 123.1, 126.1, 128.8, 131.4, 133.9, 136.8, 162.6, 164.2. HPLC-MS t_R = 1.94 minutes, 100 % purity, m/z calculated for [$C_9H_5Cl_3N_2O$ + H] = 263, found 263 (713 mg, 55 %).

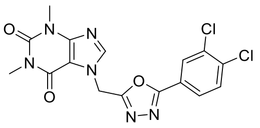

3, TH1533.

7-{[5-(3,4-dichlorophenyl)-1,3,4-oxadiazol-2-yl]methyl}-1,3-dimethyl-2,3,6,7-tetrahydro-1H-purine-2,6-dione was prepared according to General Procedure B on a 0.25 mmol scale. ^{1}H NMR (400 MHz, CHLOROFORM-*d*) δ ppm 3.41 (s, 3 H), 3.61 (s, 3 H), 5.89 (s, 2 H), 7.60 (d, *J*=8.34 Hz, 1 H), 7.81 (s, 1 H), 7.85 (dd, *J*=8.34, 2.00 Hz, 1 H), 8.10 (d, *J*=2.02 Hz, 1 H). ^{13}C NMR (101 MHz, CHLOROFORM-d) δ ppm 28.0, 29.9, 40.6, 106.7, 122.8, 126.1, 128.8, 131.4, 133.9, 137.0, 141.5, 148.8, 151.6, 155.3, 161.4, 164.2. HPLC-MS t_R = 1.70 minutes, 98.4 % purity, m/z calculated for [$C_{16}H_{12}Cl_2N_6O_3$ + H] = 407.4, found 407.1 (66 mg, 72 % yield).

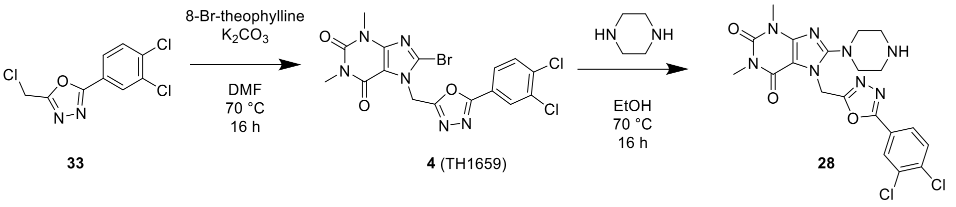

Scheme 3. Synthesis of TH5427 (**28**).

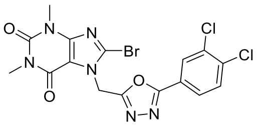

4, TH1659.

8-bromo-7-{[5-(3,4-dichlorophenyl)-1,3,4-oxadiazol-2-yl]methyl}-1,3-dimethyl-2,3,6,7-tetrahydro-1H-purine-2,6-dione was prepared according to General Procedure B on a 5 mmol scale. ^{1}H NMR (400 MHz, CHLOROFORM-*d*) δ ppm 3.41 (s, 3 H), 3.60 (s, 3 H), 5.91 (s, 2 H), 7.61 (d, *J*=8.37 Hz, 1 H), 7.86 (dd, *J*=8.37, 2.05 Hz, 1 H), 8.11 (d, *J*=1.90 Hz, 1 H). ^{13}C NMR (101 MHz, CHLOROFORM-d) δ ppm 28.1, 30.0, 41.3, 108.8, 122.9, 126.1, 128.3, 128.8, 131.3, 133.8, 136.9, 148.4, 151.1, 154.3, 160.5, 164.1. HPLC-MS t_R = 1.84 minutes, 100 % purity, m/z calculated for [$C_{16}H_{11}BrCl_2N_6O_3$ + H] = 485.0, found 485.0, (1.03 g, 44 % yield).

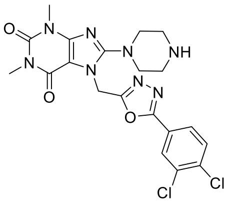

28, TH5427.

7-{[5-(3,4-dichlorophenyl)-1,3,4-oxadiazol-2-yl]methyl}-1,3-dimethyl-8-(piperazin-1-yl)-2,3,6,7-tetrahydro-1H-purine-2,6-dionewas prepared according to General Procedure C on a 0.1 mmol scale. ^{1}H NMR (400 MHz, DMSO-*d*^{6}) δ ppm 2.70 - 2.79

(m, 4 H), 3.12 - 3.21 (m, 7 H), 3.40 (s, 3 H), 5.69 (s, 2 H), 7.85 - 7.96 (m, 2 H), 8.15 (dd, J=1.90, 0.32 Hz, 1 H). ^{13}C NMR (101 MHz, DMSO-d_6) δ ppm 27.6, 29.7, 40.6, 45.1, 50.9, 104.1, 123.7, 126.8, 128.3, 132.2, 132.7, 135.2, 147.6, 151.1, 154.0, 156.8, 162.8, 163.3. HPLC-MS t_R = 1.52 minutes, 100 % purity, m/z calculated for [$C_{20}H_{20}Cl_2N_8O_3$ + H] = 491.1, found 491.2 (30.0 mg, 59 %).

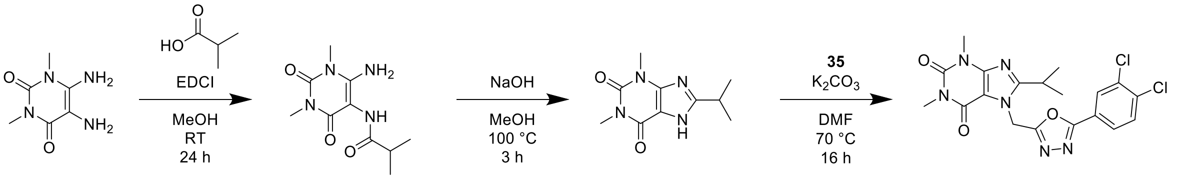

Scheme 4. Synthesis of 8-isopropyl derivative.

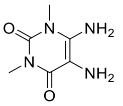

34. 5,6-diamino-1,3-dimethyl-1,2,3,4-tetrahydropyrimidine-2,4-dione was prepared as previously reported.[5]

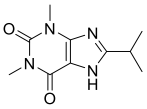

35. 1,3-dimethyl-8-(propan-2-yl)-2,3,6,7-tetrahydro-1H-purine-2,6-dione was prepared according to General Procedure D on a 0.3 mmol scale. ^{1}H NMR (400 MHz, CHLOROFORM-d) δ ppm 1.45 (d, J=7.07 Hz, 6 H), 3.23 (hept, J=6.95 Hz, 1 H), 3.48 (s, 3 H), 3.65 (s, 3 H). HPLC-MS t_R = 1.14 minutes, 98 % purity, m/z calculated for [$C_{10}H_{14}N_4O_2$+ H] = 223, found 223 (30.1 mg, 46 % yield).

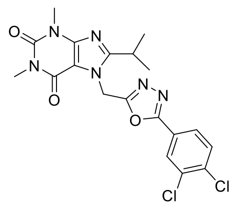

13. 7-{[5-(3,4-dichlorophenyl)-1,3,4-oxadiazol-2-yl]methyl}-1,3-dimethyl-8-(propan-2-yl)-2,3,6,7-tetrahydro-1H-purine-2,6-dione was prepared via General Procedure B on a 0.01 mmol scale.

^{1}H NMR (400 MHz, CHLOROFORM-*d*) δ ppm 1.34 (d, *J*=6.57 Hz, 6 H), 3.10 - 3.26 (m, 1 H), 3.38 (s, 3 H), 3.54 - 3.63 (m, 3 H), 5.88 (s, 2 H), 7.57 (d, *J*=8.34 Hz, 1 H), 7.76 - 7.90 (m, 1 H), 8.07 (s, 1 H). ^{13}C NMR (101 MHz, CHLOROFORM-*d*) δ ppm 21.4, 26.3, 27.9, 29.9, 38.9, 106.1, 122.9, 126.0, 128.8, 131.3, 133.8, 136.8, 148.4, 151.6, 155.3, 159.8, 161.7, 164.1. HPLC-MS t_R = 1.90 minutes, 97 % purity, m/z calculated for [$C_{19}H_{18}Cl_2N_6O_3$+ H] =449.1, found 449.1 (24.0 mg, 40 %).

Compound Characterization:

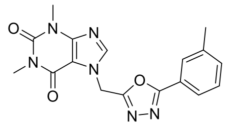

1, TH1167.

1,3-dimethyl-7-[[5-(p-tolyl)-1,3,4-oxadiazol-2-yl]methyl]purine-2,6-dione

^{1}H NMR (400 MHz, CHLOROFORM-*d*) δ ppm 2.42 - 2.44 (m, 3 H), 3.43 (s, 3 H), 3.62 (s, 3 H), 5.90 (s, 2 H), 7.34 - 7.42 (m, 2 H), 7.78 - 7.82 (m, 2 H), 7.85 (dd, *J*=1.52, 0.76 Hz, 1 H). ^{13}C NMR (101 MHz, CHLOROFORM-*d*) δ ppm 21.3, 28.0, 29.9, 40.6, 106.8, 122.9, 124.3, 127.6, 129.0, 133.1, 139.1, 141.4, 148.7, 151.6, 155.2, 160.7, 166.2. HPLC-MS t_R = 1.56 minutes, 100 % purity, m/z calculated for [$C_{17}H_{16}N_6O_3$ + H] = 353.1, found 353.2.

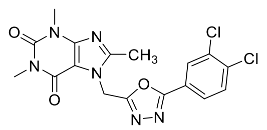

5. 7-{[5-(3,4-dichlorophenyl)-1,3,4-oxadiazol-2-yl]methyl}-1,3,8-trimethyl-2,3,6,7-tetrahydro-1H-purine-2,6-dione

^{1}H NMR (400 MHz, CHLOROFORM-*d*) δ ppm 2.61 (s, 3 H), 3.43 (s, 3 H), 3.61 (s, 3 H), 5.89 (s, 2 H), 7.61 (d, *J*=8.37 Hz, 1 H), 7.86 (dd, *J*=8.37, 2.05 Hz, 1 H), 8.12 (d, *J*=1.90 Hz, 1 H). ^{13}C NMR (101 MHz, CHLOROFORM-*d*) δ ppm 13.3, 27.9, 29.8, 39.3, 106.6, 122.8, 126.1, 128.8, 131.4, 133.8, 135.4, 136.9, 148.2, 151.5, 155.1, 161.2, 164.2. HPLC-MS t_R = 1.72 minutes, 93.9 % purity, m/z calculated for [$C_{17}H_{14}Cl_2N_6O_3$ + H] = 421.1, found 421.2.

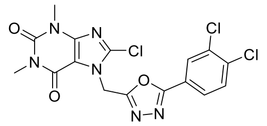

6. 8-chloro-7-{[5-(3,4-dichlorophenyl)-1,3,4-oxadiazol-2-yl]methyl}-1,3-dimethyl-2,3,6,7-tetrahydro-1H-purine-2,6-dione

^{1}H NMR (400 MHz, CHLOROFORM-*d*) δ ppm 3.40 (s, 3 H), 3.54 - 3.64 (m, 3 H), 5.90 (s, 2 H), 7.61 (d, *J*=8.37 Hz, 1 H), 7.86 (dd, *J*=8.37, 2.05 Hz, 1 H), 8.11 (d, *J*=2.05 Hz, 1 H). ^{13}C NMR (101 MHz, CHLOROFORM-*d*) δ ppm 28.1, 30.0, 40.2, 107.6, 122.8, 126.1, 128.8, 131.4, 133.8, 136.9, 139.3, 147.4, 151.1, 154.4, 160.4, 164.2. HPLC-MS t_R = 1.84 minutes, 100 % purity, m/z calculated for [$C_{16}H_{11}Cl_3N_6O_3$+ H] = 441.0, found 441.0.

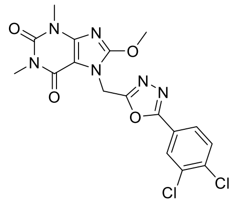

7. 7-{[5-(3,4-dichlorophenyl)-1,3,4-oxadiazol-2-yl]methyl}-8-methoxy-1,3-dimethyl-2,3,6,7-tetrahydro-1H-purine-2,6-dione

^{1}H NMR (400 MHz, DMSO-d^{6}) δ ppm 3.18 (s, 3 H), 3.41 (s, 3 H), 4.14 (s, 3 H), 5.64 (s, 2 H), 7.85 - 7.95 (m, 2 H), 8.13 (s, 1 H). ^{13}C NMR (101 MHz, CHLOROFORM-*d*) δ ppm 27.8, 29.9, 37.8, 58.4, 102.7, 123.1, 126.0, 128.7, 131.3, 133.8, 136.6, 146.7, 151.6, 154.7, 156.3, 161.6, 163.9. HPLC-MS t_R = 1.81 minutes, 95.0 % purity, m/z calculated for [$C_{17}H_{14}Cl_2N_6O_4$ + H] = 437.1, found 437.1.

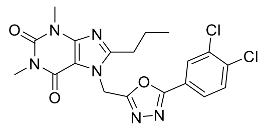

8. 7-{[5-(3,4-dichlorophenyl)-1,3,4-oxadiazol-2-yl]methyl}-1,3-dimethyl-8-propyl-2,3,6,7-tetrahydro-1H-purine-2,6-dione

^{1}H NMR (400 MHz, CHLOROFORM-*d*) δ ppm 0.98 (t, *J*=7.45 Hz, 3 H), 1.72 - 1.92 (m, 2 H), 2.77 (t, *J*=7.58 Hz, 2 H), 3.36 (s, 3 H), 3.55 (s, 3 H), 5.85 (s, 2 H), 7.54 (d, *J*=8.34 Hz, 1 H), 7.79 (dd, *J*=8.34, 1.77 Hz, 1 H), 8.03 (d, *J*=1.77 Hz, 1 H). ^{13}C NMR (101 MHz, CHLOROFORM-*d*) δ ppm 13.8, 21.1, 27.9, 28.7, 29.8, 39.1, 106.4, 122.9, 126.1, 128.8, 131.4, 133.8, 136.9, 148.4, 151.6, 155.1, 155.2, 161.5, 164.1. HPLC-MS t_R = 1.89 minutes, 100 % purity, m/z calculated for [$C_{20}H_{20}Cl_2N_6O_3$ + H] = 449.1, found 449.2.

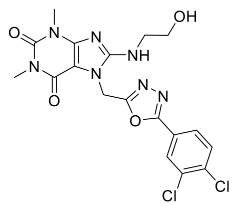

9. 7-{[5-(3,4-dichlorophenyl)-1,3,4-oxadiazol-2-yl]methyl}-8-[(2-hydroxyethyl)amino]-1,3-dimethyl-2,3,6,7-tetrahydro-1H-purine-2,6-dione

^{1}H NMR (400 MHz, DMSO-*d*^{6}) δ ppm 3.14 (s, 3 H), 3.38 (s, 3 H), 3.43 (q, J = 6.1 Hz, 2 H), 3.56 (q, J = 5.7 Hz, 2 H), 4.77 (t, J = 6.0 Hz, 1 H), 5.68 (s, 2 H), 7.44 (t, J = 5.5 Hz, 1 H), 7.88 - 7.96 (m, 2 H), 8.18 (d, J = 1.7 Hz, 1 H). ^{13}C NMR (101 MHz, CHLOROFORM-*d*) d ppm 27.8, 29.9, 37.3, 46.1, 62.3, 102.2, 122.6, 126.1, 128.8, 131.4, 133.9, 137.1, 148.2, 151.5, 154.1, 154.3, 161.7, 164.3. HPLC-MS t_R = 1.41 minutes, 100 % purity, m/z calculated for [$C_{18}H_{17}Cl_2N_7O_4$+ H] = 466.1, found 466.1.

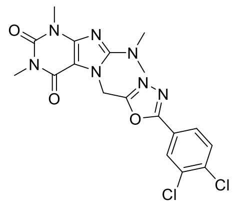

10. 7-{[5-(3,4-dichlorophenyl)-1,3,4-oxadiazol-2-yl]methyl}-8-(dimethylamino)-1,3-dimethyl-2,3,6,7-tetrahydro-1H-purine-2,6-dione

^{1}H NMR (400 MHz, CHLOROFORM-*d*) δ ppm 3.04 (s, 6 H), 3.37 (s, 3 H), 3.56 (s, 3 H), 5.71 (s, 2 H), 7.59 (d, *J*=8.34 Hz, 1 H), 7.84 (dd, *J*=8.34, 2.02 Hz, 1 H), 8.10 (d, *J*=2.02 Hz, 1 H). ^{13}C NMR (101 MHz, CHLOROFORM-*d*) δ ppm 27.8, 29.8, 40.4, 41.9, 104.1, 123.1, 126.0, 128.8, 131.3, 133.8, 136.7, 148.3, 151.7, 154.6, 157.9, 162.2, 163.9. HPLC-MS t_R = 1.81 minutes, 100 % purity, m/z calculated for [$C_{18}H_{17}Cl_2N_7O_3$+ H] =450.1, found 450.1.

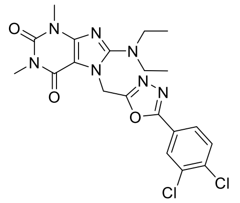

11. 7-{[5-(3,4-dichlorophenyl)-1,3,4-oxadiazol-2-yl]methyl}-8-(diethylamino)-1,3-dimethyl-2,3,6,7-tetrahydro-1H-purine-2,6-dione

^{1}H NMR (400 MHz, CHLOROFORM-*d*) δ ppm 2.36 (s, 3 H), 2.57 (br. s., 4 H), 3.37 (s, 6 H), 3.57 (s, 3 H), 5.64 (s, 2 H), 7.60 (d, *J*=8.34 Hz, 1 H), 7.79 - 7.93 (m, 1 H), 8.11 (d, *J*=1.77 Hz, 1 H). ^{13}C NMR (101 MHz, CHLOROFORM-*d*) δ ppm 13.0, 27.7, 29.8, 40.1, 46.3, 104.2, 123.1, 126.0, 128.7, 131.3, 133.7, 136.6, 148.3, 151.7, 154.6, 156.7, 162.2, 163.7. HPLC-MS t_R = 1.98 minutes, 96.9 % purity, m/z calculated for [$C_{20}H_{21}Cl_2N_7O_3$+ H] =478.1, found 478.2.

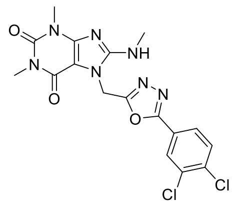

12. 7-{[5-(3,4-dichlorophenyl)-1,3,4-oxadiazol-2-yl]methyl}-1,3-dimethyl-8-(methylamino)-2,3,6,7-tetrahydro-1H-purine-2,6-dione

^{1}H NMR (400 MHz, DMSO-*d*^{6}) δ ppm 2.92 (d, *J*=4.58 Hz, 3 H), 3.14 (s, 3 H), 3.40 (s, 3 H), 5.64 (s, 2 H), 7.36 (q, *J*=4.53 Hz, 1 H), 7.86 - 7.96 (m, 2 H), 8.10 - 8.19 (m, 1 H). ^{13}C NMR could not be obtained due to poor solubility. HPLC-MS t_R = 1.70 minutes, 93.5 % purity, m/z calculated for [$C_{17}H_{15}Cl_2N_7O_3$+ H] =436, found 436.1.

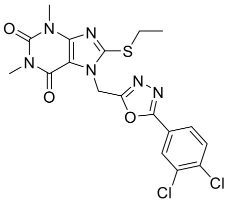

14. 7-{[5-(3,4-dichlorophenyl)-1,3,4-oxadiazol-2-yl]methyl}-8-(ethylsulfanyl)-1,3-dimethyl-2,3,6,7-tetrahydro-1H-purine-2,6-dione

^{1}H NMR (400 MHz, CHLOROFORM-*d*) δ ppm 1.43 (t, *J*=7.35 Hz, 3 H), 3.32 (q, *J*=7.42 Hz, 2 H), 3.40 (s, 3 H), 3.60 (s, 3 H), 5.83 (s, 2 H), 7.59 (d, *J*=8.53 Hz, 1 H), 7.86 (dd, *J*=8.45, 1.97 Hz, 1 H), 8.11 (d, *J*=1.90 Hz, 1 H). ^{13}C NMR (101 MHz, CHLOROFORM-*d*) δ ppm 14.9, 27.9, 28.0, 29.9, 39.9, 107.8, 123.0, 126.1, 128.8, 131.3, 133.8, 136.7, 140.8, 148.9, 151.4, 154.4, 161.1, 164.0. HPLC-MS t_R = 1.96 minutes, 100 % purity, m/z calculated for [$C_{18}H_{16}Cl_2N_6O_3S$+ H] = 467.1, found 467.1.

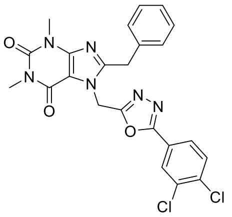

15. 8-benzyl-7-{[5-(3,4-dichlorophenyl)-1,3,4-oxadiazol-2-yl]methyl}-1,3-dimethyl-2,3,6,7-tetrahydro-1H-purine-2,6-dione

^{1}H NMR (400 MHz, CHLOROFORM-*d*) δ ppm 3.39 (s, 3 H), 3.63 (s, 3 H), 4.28 (s, 2 H), 5.73 (s, 2 H), 7.10 - 7.22 (m, 3 H), 7.22 - 7.29 (m, 2 H), 7.56 (d, *J*=8.34 Hz, 1 H), 7.74 (dd, *J*=8.34, 2.02 Hz, 1 H), 7.97 (d, *J*=2.02 Hz, 1 H). ^{13}C NMR (101 MHz, CHLOROFORM-*d*) δ ppm 28.0, 29.9, 33.6, 39.3, 107.1, 122.8, 126.0, 127.5, 128.3, 128.7, 129.1, 131.3, 133.7, 134.3, 136.7, 148.1, 151.5, 152.9, 155.2, 161.2, 163.9. HPLC-MS t_R = 1.94 minutes, 97.2 % purity, m/z calculated for [$C_{23}H_{18}Cl_2N_6O_3$+ H] =497.1, found 497.1.

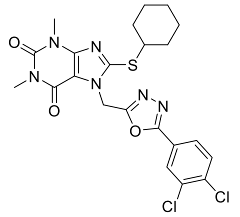

16. 8-(cyclohexylsulfanyl)-7-{[5-(3,4-dichlorophenyl)-1,3,4-oxadiazol-2-yl]methyl}-1,3-dimethyl-2,3,6,7-tetrahydro-1H-purine-2,6-dione

^{1}H NMR (400 MHz, CHLOROFORM-*d*) δ ppm 1.27 - 1.35 (m, 1 H), 1.37 - 1.64 (m, 5 H), 1.77 (dt, *J*=13.11, 3.95 Hz, 2 H), 2.01 - 2.16 (m, 2 H), 3.39 (s, 3 H), 3.61 (s, 3 H), 3.76 - 3.92 (m, 1 H), 5.85 (s, 2 H), 7.53 - 7.65 (m, 1 H), 7.86 (dd, *J*=8.45, 1.97 Hz, 1 H), 8.10 (d, *J*=1.90 Hz, 1 H). ^{13}C NMR (101 MHz, CHLOROFORM-*d*) δ ppm 25.4, 25.8, 28.0, 29.9, 33.4, 40.1, 48.3, 107.7, 123.1, 126.1, 128.8, 131.3, 133.8, 136.7, 148.9, 151.5, 151.5, 154.5, 161.3, 163.9. HPLC-MS t_R = 2.18 minutes, 98.8 % purity, m/z calculated for [$C_{22}H_{22}Cl_2N_6O_3S$ + H] = 521.1, found 521.2.

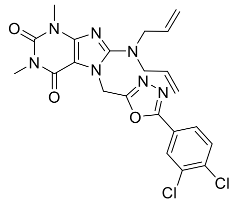

17. 8-[bis(prop-2-en-1-yl)amino]-7-{[5-(3,4-dichlorophenyl)-1,3,4-oxadiazol-2-yl]methyl}-1,3-dimethyl-2,3,6,7-tetrahydro-1H-purine-2,6-dione

^{1}H NMR (400 MHz, CHLOROFORM-*d*) δ ppm 3.35 (s, 3 H), 3.55 (s, 3 H), 3.94 (dt, *J*=5.69, 1.26 Hz, 4 H), 5.17 - 5.29 (m, 4 H), 5.66 (s, 2 H), 5.80 - 5.94 (m, 2 H), 7.58 (d, *J*=8.53 Hz, 1 H), 7.81 - 7.89 (m, 1 H), 8.09 (d, *J*=1.90 Hz, 1 H). HPLC-MS t_R = 1.72 minutes, 96.2 % purity, m/z calculated for [$C_{22}H_{21}Cl_2N_7O_3$+ H] = 502.1, found 502.2.

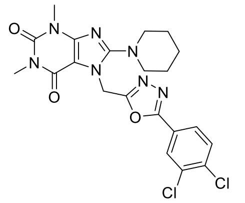

18. 7-{[5-(3,4-dichlorophenyl)-1,3,4-oxadiazol-2-yl]methyl}-1,3-dimethyl-8-(piperidin-1-yl)-2,3,6,7-tetrahydro-1H-purine-2,6-dione

^{1}H NMR (400 MHz, CHLOROFORM-*d*) δ ppm 1.58 - 1.71 (m, 6 H), 3.20 - 3.29 (m, 4 H), 3.37 (s, 3 H), 3.61 (s, 3 H), 5.63 (s, 2 H), 7.60 (d, *J*=8.37 Hz, 1 H), 7.86 (dd, *J*=8.45, 1.97 Hz, 1 H), 8.11 (d, *J*=2.05 Hz, 1 H). ^{13}C NMR (101 MHz, CHLOROFORM-*d*) d ppm 23.8, 25.4, 27.8, 29.8, 40.3, 51.6, 104.5, 123.1, 126.0, 128.7, 131.3, 133.8, 136.6, 148.1, 151.7, 154.8, 157.5, 162.2, 163.8. HPLC-MS t_R = 2.00 minutes, 100 % purity, m/z calculated for [$C_{21}H_{21}Cl_2N_7O_3$ + H] = 490.1, found 490.2.

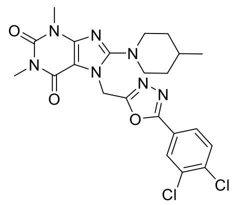

19. 7-{[5-(3,4-dichlorophenyl)-1,3,4-oxadiazol-2-yl]methyl}-1,3-dimethyl-8-(4-methylpiperidin-1-yl)-2,3,6,7-tetrahydro-1H-purine-2,6-dione

^{1}H NMR (400 MHz, CHLOROFORM-*d*) δ ppm 0.98 (d, *J*=6.63 Hz, 3 H), 1.30 (qd, *J*=12.32, 3.95 Hz, 2 H), 1.57 (ddd, *J*=11.06, 6.95, 3.79 Hz, 1 H), 1.73 (dd, *J*=12.72, 2.29 Hz, 2 H), 3.02 (td, *J*=12.48, 2.53 Hz, 2 H), 3.37 (s, 3 H), 3.51 (d, *J*=12.48 Hz, 2 H), 3.56 - 3.62 (m, 3 H), 5.63 (s, 2 H), 7.60 (d, *J*=8.37 Hz, 1 H), 7.86 (dd, *J*=8.37, 2.05 Hz, 1 H), 8.11 (d, *J*=2.05 Hz, 1 H). ^{13}C NMR (101 MHz, CHLOROFORM-*d*) δ ppm 21.7, 27.8, 29.8, 30.4, 33.6, 40.3, 51.0, 104.5, 123.1, 126.0, 128.7, 131.3,

133.8, 136.6, 148.1, 151.7, 154.8, 157.5, 162.2, 163.8. HPLC-MS t_R = 2.07 minutes, 100 % purity, m/z calculated for [$C_{22}H_{23}Cl_2N_7O_3$ + H] = 504.1, found 504.3.

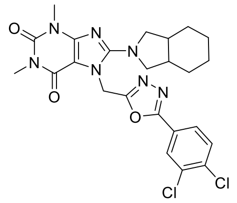

20. 7-{[5-(3,4-dichlorophenyl)-1,3,4-oxadiazol-2-yl]methyl}-1,3-dimethyl-8-(octahydro-1H-isoindol-2-yl)-2,3,6,7-tetrahydro-1H-purine-2,6-dione

^{1}H NMR (400 MHz, DMSO-d^6) δ ppm 1.29 - 1.59 (m, 8 H), 2.17 - 2.30 (m, 2 H), 3.15 (s, 3 H), 3.36 (s, 3 H), 3.52 - 3.55 (m, 2 H), 3.62 -3.66 (m, 2 H), 5.86 (s, 2 H), 7.85 - 7.94 (m, 2 H), 8.06 - 8.15 (m, 1 H). ^{13}C NMR (101 MHz, CHLOROFORM-*d*) d ppm 22.6, 25.6, 27.7, 29.8, 37.3, 39.7, 53.8, 102.9, 123.0, 126.0, 128.8, 131.3, 133.8, 136.7, 149.3, 151.8, 154.2, 155.4, 162.5, 163.9. HPLC-MS t_R = 1.88 minutes, 100 % purity, m/z calculated for [$C_{24}H_{25}Cl_2N_7O_3$ + H] = 530.1, found 530.2.

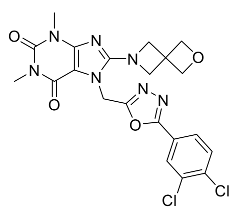

21. 7-{[5-(3,4-dichlorophenyl)-1,3,4-oxadiazol-2-yl]methyl}-1,3-dimethyl-8-{2-oxa-6-azaspiro[3.3]heptan-6-yl}-2,3,6,7-tetrahydro-1H-purine-2,6-dione

^{1}H NMR (400 MHz ,DMSO-d^6) δ ppm 3.15 (s, 3 H), 3.37 (s, 3H), 4.42 (s, 4 H), 4.69 (s, 4 H), 5.61 (s, 2 H), 7.91 (q, J = 8.1 Hz, 2 H), 8.15 (s, 1 H). ^{13}C NMR could not be obtained due to poor solubility. HPLC-MS t_R = 1.70 minutes, 98,0 % purity, m/z calculated for [$C_{21}H_{19}Cl_2N_7O_4$+ H] = 504.1, found 504.2.

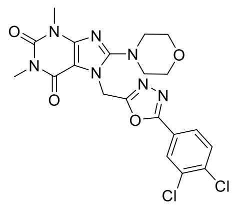

22. 7-{[5-(3,4-dichlorophenyl)-1,3,4-oxadiazol-2-yl]methyl}-1,3-dimethyl-8-(morpholin-4-yl)-2,3,6,7-tetrahydro-1H-purine-2,6-dione

^{1}H NMR (400 MHz, CHLOROFORM-*d*) δ ppm 3.20 - 3.34 (m, 4 H), 3.38 (s, 3 H), 3.57 (s, 3 H), 3.74 - 3.92 (m, 4 H), 5.68 (s, 2 H), 7.60 (d, *J*=8.37 Hz, 1 H), 7.86 (dd, *J*=8.45, 1.97 Hz, 1 H), 8.11 (d, *J*=1.90 Hz, 1 H). ^{13}C NMR (101 MHz, CHLOROFORM-*d*) δ ppm 28.2, 30.2, 40.3, 51.0, 66.5, 105.1, 123.3, 126.3, 129.1, 131.7, 134.1, 137.1, 148.1, 151.9, 155.2, 156.4, 162.3, 164.3. HPLC-MS t_R = 1.79 minutes, 100 % purity, m/z calculated for [$C_{20}H_{19}Cl_2N_7O_4$+ H] = 492.1, found 492.2.

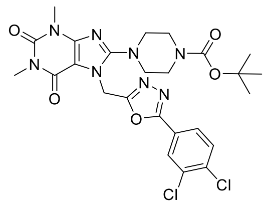

23. tert-butyl 4-(7-{[5-(3,4-dichlorophenyl)-1,3,4-oxadiazol-2-yl]methyl}-1,3-dimethyl-2,6-dioxo-2,3,6,7-tetrahydro-1H-purin-8-yl)piperazine-1-carboxylate

^{1}H NMR (400 MHz, CHLOROFORM-*d*) δ ppm 1.47 (s, 9 H), 3.18 - 3.31 (m, 4 H), 3.38 (s, 3 H), 3.48 - 3.63 (m, 7 H), 5.68 (s, 2 H), 7.55 - 7.65 (m, 1 H), 7.87 (dd, *J*=8.37, 2.05 Hz, 1 H), 8.12 (d, *J*=1.90 Hz, 1 H). ^{13}C NMR (101 MHz, CHLOROFORM-*d*) d ppm 27.8, 28.4, 29.9, 40.0, 50.4, 50.4, 80.3, 104.8, 123.0, 126.0, 128.8, 131.4, 133.8, 136.8, 147.7, 151.6, 154.5, 154.9, 156.1, 161.9, 164.0. HPLC-MS t_R = 2.02 minutes, 100 % purity, m/z calculated for [$C_{25}H_{28}Cl_2N_8O_5$+ H] =591.2, found 591.3.

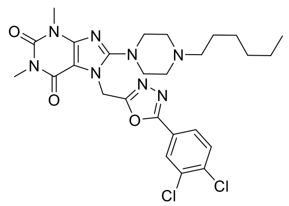

24. 7-{[5-(3,4-dichlorophenyl)-1,3,4-oxadiazol-2-yl]methyl}-8-(4-hexylpiperazin-1-yl)-1,3-dimethyl-2,3,6,7-tetrahydro-1H-purine-2,6-dione

^{1}H NMR (400 MHz, CHLOROFORM-*d*) δ ppm 0.82 - 0.95 (m, 3 H), 1.30 (br. s., 6 H), 1.56 (br. s., 2 H), 2.48 (br. s., 2 H), 2.69 (br. s., 4 H), 3.37 (s, 3 H), 3.44 (br. s., 4 H), 3.56 (s, 3 H), 5.64 (s, 2 H), 7.60 (d, *J*=8.37 Hz, 1 H), 7.86 (dd, *J*=8.37, 2.05 Hz, 1 H), 8.11 (d, *J*=1.90 Hz, 1 H). ^{13}C NMR (101 MHz, CHLOROFORM-*d*) d ppm 14.0, 22.5, 27.0, 27.8, 29.9, 31.6, 40.2, 49.9 (br), 52.1, 58.5, 104.7, 123.0, 126.0, 128.8, 131.3, 133.8, 136.7, 147.8, 151.6, 154.8, 157.3, 162.0, 163.9. HPLC-MS t_R = 1.78 minutes, 100 % purity, m/z calculated for [$C_{26}H_{32}Cl_2N_8O_3$ + H] = 575.2, found 575.3.

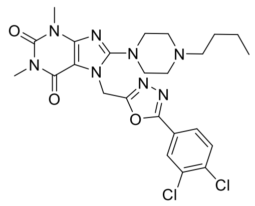

25, TH5423.

8-(4-butylpiperazin-1-yl)-7-{[5-(3,4-dichlorophenyl)-1,3,4-oxadiazol-2-yl]methyl}-1,3-dimethyl-2,3,6,7-tetrahydro-1H-purine-2,6-dione

^{1}H NMR (400 MHz, CHLOROFORM-*d*) δ ppm 0.96 (t, *J*=7.35 Hz, 3 H), 1.38 (dq, *J*=14.89, 7.41 Hz, 2 H), 1.67 (br. s., 4 H), 2.67 (br. s., 2 H), 2.91 (br. s., 2 H), 3.37 (s, 3 H), 3.43 - 3.65 (m, 7 H), 5.65 (s, 2 H), 7.61 (d, *J*=8.37 Hz, 1 H), 7.87 (dd, *J*=8.37, 2.05 Hz, 1 H) 8.12 (d, *J*=1.90 Hz, 1 H). ^{13}C NMR (101 MHz, DMSO-d_6) δ ppm 13.9, 16.6, 20.1, 27.4, 28.2, 29.5, 49.7, 52.1, 57.4, 103.9, 123.5, 126.6, 128.1, 132.0,

132.4, 135.0, 147.3, 150.9, 153.8, 156.3, 162.6, 163.1. HPLC-MS t_R = 1.66 minutes, 98 % purity, m/z calculated for [$C_{24}H_{28}Cl_2N_8O_3$ + H] = 547.2, found 547.3.

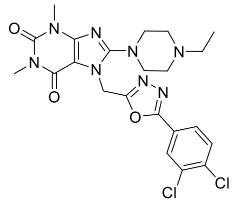

26, TH5424.

7-{[5-(3,4-dichlorophenyl)-1,3,4-oxadiazol-2-yl]methyl}-8-(4-ethylpiperazin-1-yl)-1,3-dimethyl-2,3,6,7-tetrahydro-1H-purine-2,6-dione

^{1}H NMR (400 MHz, CHLOROFORM-*d*) δ ppm 1.26 (br. s., 3 H), 2.71 (br. s., 2 H), 2.77 - 2.98 (m, 4 H), 3.37 (s, 3 H), 3.56 (s, 7 H), 5.65 (s, 2 H), 7.61 (d, *J*=8.37 Hz, 1 H), 7.87 (dd, *J*=8.45, 1.97 Hz, 1 H), 8.12 (d, *J*=1.90 Hz, 1 H). ^{13}C NMR (101 MHz, DMSO-d_6) δ ppm 11.8, 27.4, 29.6, 49.7, 51.6, 51.7 (br), 104.0, 123.5, 126.6, 128.1, 132.0, 132.4, 135.0, 147.3, 150.9, 153.8, 156.3, 162.6, 163.1. HPLC-MS t_R = 1.56 minutes, 98 % purity, m/z calculated for [$C_{22}H_{24}Cl_2N_8O_3$ + H] = 519.1, found 519.2.

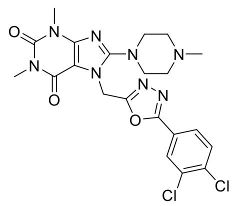

27. 7-{[5-(3,4-dichlorophenyl)-1,3,4-oxadiazol-2-yl]methyl}-1,3-dimethyl-8-(4-methylpiperazin-1-yl)-2,3,6,7-tetrahydro-1H-purine-2,6-dione

^{1}H NMR (400 MHz, CHLOROFORM-*d*) δ ppm 2.56 (br. s., 3 H), 2.88 (br. s., 4 H), 3.37 (s, 3 H), 3.49 - 3.65 (m, 7 H), 5.65 (s, 2 H), 7.61 (d, *J*=8.37 Hz, 1 H), 7.87 (dd, *J*=8.45, 1.97 Hz, 1 H), 8.12 (d, *J*=1.90 Hz, 1 H). ^{13}C NMR (101 MHz, DMSO-d_6) d ppm 27.4, 29.5, 45.7, 49.5, 53.9, 103.9, 123.5, 126.6, 128.1, 132.0, 132.4, 135.0,

147.3, 150.9, 153.8, 156.3, 162.6, 163.1. HPLC-MS t_R = 1.54 minutes, 98.4 % purity, m/z calculated for [$C_{21}H_{22}Cl_2N_8O_3$ + H] = 505.1, found 505.2.

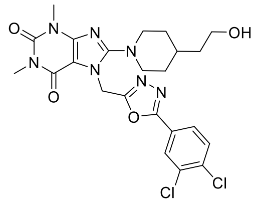

29. 7-{[5-(3,4-dichlorophenyl)-1,3,4-oxadiazol-2-yl]methyl}-8-[4-(2-hydroxyethyl)piperidin-1-yl]-1,3-dimethyl-2,3,6,7-tetrahydro-1H-purine-2,6-dione

^{1}H NMR (400 MHz, DMSO-d^6) δ ppm 1.13 - 1.27 (m, 2 H), 1.35 (q, J = 6.5 Hz, 2 H), 1.49 - 1.62 (m, 1 H), 1.64 - 1.74 (m, 2 H), 2.86 - 2.99 (m, 2 H), 3.40 (s, 3 H), 3.14 (s, 3 H), 3.41 - 3.46 (m, 2 H), 3.55 (d, J = 12.6 Hz, 2 H), 4.37 (t, J = 5.1 Hz, 1 H), 5.66 (s, 2 H), 7.84 - 7.98 (m, 2 H), 8.14 (d, J = 1.7 Hz, 1 H). ^{13}C NMR (101 MHz, DMSO-d_6) d ppm 27.4, 29.5, 31.4, 31.5, 40.2, 40.4, 50.2, 58.1, 103.8, 123.5, 126.6, 128.1, 132.0, 132.4, 135.0, 147.5, 150.9, 153.7, 156.9, 162.6, 163.2. HPLC-MS t_R = 1.76 minutes, 99.3 % purity, m/z calculated for [$C_{23}H_{25}Cl_2N_7O_4$+ H] = 534.1, found 534.2.

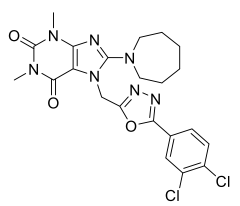

30. 8-(azepan-1-yl)-7-{[5-(3,4-dichlorophenyl)-1,3,4-oxadiazol-2-yl]methyl}-1,3-dimethyl-2,3,6,7-tetrahydro-1H-purine-2,6-dione

^{1}H NMR (400MHz ,DMSO-d^6) δ ppm 1.59 - 1.46 (m, 4 H) , 1.80 - 1.67 (m, 4 H) , 3.15 (s, 3 H), 3.38 (s, 3 H), 3.61 - 3.51 (m, 4 H) , 5.75 (s, 2 H) , 7.94 - 7.83 (m, 2 H), 8.12 (d, J = 1.6 Hz, 1 H). ^{13}C NMR (101 MHz, CHLOROFORM-d) d ppm 27.2, 27.7, 28.5, 29.8, 40.7, 51.8, 103.6, 123.1, 126.0, 128.7, 131.3, 133.7, 136.6, 148.9, 151.8,

154.4, 158.0, 162.4, 163.8. HPLC-MS t_R = 2.04 minutes, 100 % purity, m/z calculated for $[C_{22}H_{23}Cl_2N_7O_3 + H]$ = 504.1, found 504.2.

Supplementary References

1. Lipinski, C.A. Lead- and drug-like compounds: the rule-of-five revolution. *Drug Discov Today Technol* **1**, 337-341 (2004).
2. Almqvist, H.*, et al.* CETSA screening identifies known and novel thymidylate synthase inhibitors and slow intracellular activation of 5-fluorouracil. *Nat Commun* **7**, 11040 (2016).
3. Baykov, A.A., Evtushenko, O.A. & Avaeva, S.M. A malachite green procedure for orthophosphate determination and its use in alkaline phosphatase-based enzyme immunoassay. *Anal Biochem* **171**, 266-270 (1988).
4. MERLOS, M.*, et al.* STRUCTURE-ACTIVITY-RELATIONSHIPS IN A SERIES OF XANTHINE DERIVATIVES WITH ANTIBRONCHOCONSTRICTORY AND BRONCHODILATORY ACTIVITIES. *European Journal of Medicinal Chemistry* **25**, 653-658 (1990).
5. Erickson, R.H.*, et al.* 1,3,8-trisubstituted xanthines. Effects of substitution pattern upon adenosine receptor A1/A2 affinity. *J Med Chem* **34**, 1431-1435 (1991).
